# Proteomic signatures of COVID-19 Post-Vaccination/Post-Infection Syndrome (PV/PIS): insights into immune dysregulation and coagulopathy

**DOI:** 10.3389/fcimb.2026.1753348

**Published:** 2026-03-25

**Authors:** Maxine Waters, Mare Vlok, Elouise E. Kroon, Maritha J. Kotze, Kelebogile E. Moremi, Sunday O. Oladejo, Kanshukan Rajaratnam, Jean M. Nunes, Chantelle Venter, Chantelle J. Scott, Douglas B. Kell, Etheresia Pretorius

**Affiliations:** 1 Department of Physiological Sciences, Faculty of Science, Stellenbosch University, Stellenbosch, South Africa; 2 Trace Labs (PTY) LTD, Loevenstein, Cape Town, South Africa; 3 South African Medical Research Council Centre for Tuberculosis Research, Division of Molecular Biology and Human Genetics, Faculty of Medicine and Health Sciences, Stellenbosch University, Cape Town, South Africa; 4 Division of Chemical Pathology, Department of Pathology, Faculty of Medicine and Health Sciences, Stellenbosch University, Francie Van Zijl Drive, Tygerberg, Cape Town, South Africa; 5 Division of Chemical Pathology, Department of Pathology, National Health Laboratory Service, Tygerberg Hospital, Cape Town, South Africa; 6 School for Data Science and Computational Thinking, Stellenbosch University, Stellenbosch, South Africa; 7 Department of Biochemistry, Cell and Systems Biology, Institute of Systems, Molecular and Integrative Biology, Faculty of Health and Life Sciences, University of Liverpool, Liverpool, United Kingdom; 8 The Novo Nordisk Foundation Centre for Biosustainability, Technical University of Denmark, Kongens Lyngby, Denmark

**Keywords:** heterogenous amyloid deposits (microclots), inflammatory molecules, Long COVID, proteomics, PV/PIS

## Abstract

**Introduction:**

During the global rollout of COVID-19 vaccines, a subset of individuals reported persistent symptoms following vaccination, with clinical presentations overlapping those of Long COVID and requiring individualised treatment strategies. Distinguishing between vaccine-related adverse events and post-infectious sequelae is challenging, particularly given the potential for unrecognised asymptomatic or mild SARS-CoV-2 infection before or after vaccination. To address this complexity, we defined our disease cohort as individuals experiencing persistent symptoms ( ≥ 12 weeks) following SARS-CoV-2 vaccination, without a confirmed history of prolonged symptoms after acute infection; for clarity, we refer to this group as presenting with Post-Vaccination/Post-Infection Syndrome (PV/PIS).

**Methods:**

In this study, we conducted a plasma proteomic analysis of digested microclot deposits isolated from platelet-poor plasma samples of 14 individuals with PV/PIS compared to 16 healthy controls, using liquid-chromatography-mass spectrometry.

**Results:**

We identified significant alterations in coagulation factors, acute phase proteins, and immune response modulators in the PV/PIS group compared to controls. Notably, elevated levels of serum amyloid A1 and A2, attractin, and coagulation factors X and XI were observed, alongside downregulation of immune-regulatory proteins. These findings suggest that PV/PIS is characterised by persistent immune dysregulation and coagulopathy.

**Conclusions:**

This proteomic signature was found to only partially overlap with that previously reported in a proteomics analysis on Long COVID samples, collected prior to vaccination availability. Our results highlight the complex interplay between immune activation, endothelial dysfunction, and coagulation pathologies in PV/PIS, with distinct differences detected between these systems in Long COVID and PV/PIS, paving the way for more targeted protein research in these conditions.

## Graphical Abstract

**Figure:**
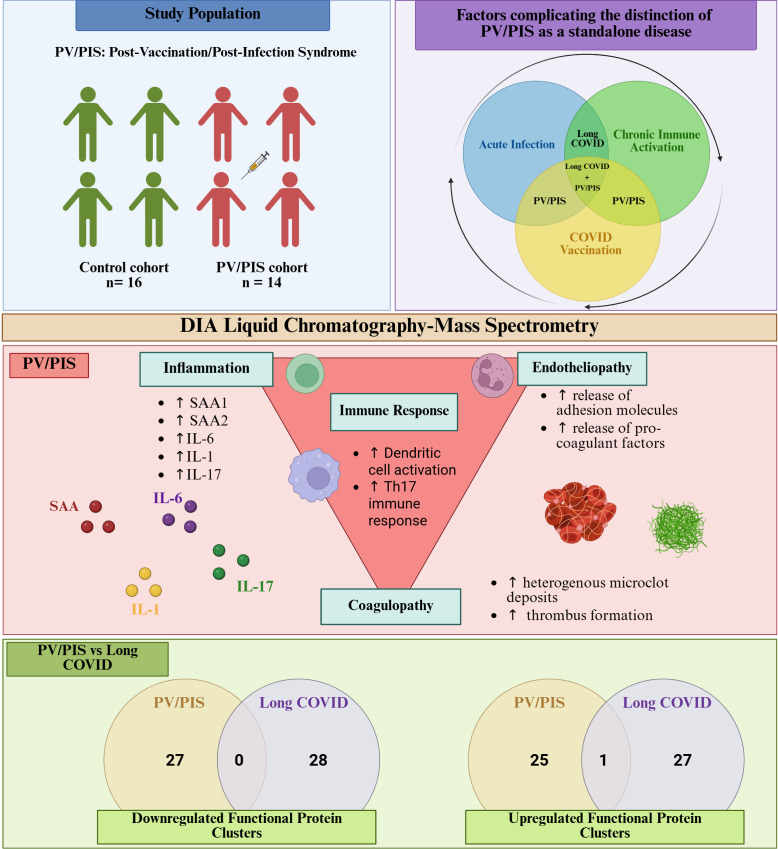
Created in https://BioRender.com

## 1 Introduction

Post-acute sequelae of COVID-19 (PASC), commonly known as Long COVID, is estimated to have affected over 400 million individuals worldwide, contributing to an annual economic burden of $1 trillion – representing approximately 1% of the global economy (Al-Aly et al., 2024). Initially recognised by affected individuals themselves and reported via social media and various community forums (Callard and Perego, 2021), Long COVID has since been acknowledged as a potentially disabling condition by the Centres for Disease Control and Prevention (CDC) (CDC, 2024). Ewing et al. (2025) published expert consensus recommendations for physicians, emphasising the need for individualised treatment in cases where vaccination exacerbated Long COVID symptoms or resulted in adverse vaccine side effects (Ewing et al., 2025).

The World Health Organization (WHO) defines Long COVID as the persistence or emergence of new symptoms beyond two months after an acute SARS-CoV-2 infection, with no alternative explanation (WHO, 2022). This condition affects multiple physiological systems and presents with a broad spectrum of symptoms, including neurological (brain fog, extreme fatigue, sleep disturbances), cardiovascular (orthostatic intolerance, chest pain, shortness of breath, tachycardia), musculoskeletal (joint pain, muscle weakness), and psychosocial manifestations (depression, anxiety, social isolation) (Wiersinga et al., 2020; Campen et al., 2021; Raveendran, 2021; Monaghan et al., 2022; Subramanian et al., 2022; van Campen and Visser, 2022). Notably, more than 200 symptoms have been linked to Long COVID globally, with affected individuals experiencing varying combinations of symptoms and/or cyclic patterns of symptom recurrence (Davis et al., 2021; CDC, 2024). Symptom severity and duration of Long COVID are not always dependent on a patient’s hospitalisation for acute COVID. Severe cases have been observed in both patients who were hospitalised for acute COVID (Abu Hamdh and Nazzal, 2023; Natarajan et al., 2023) and in those who were not (Subramanian et al., 2022). Long COVID is a complex condition resulting from various influences, including host-specific factors (e.g. co-morbidities), viral dynamics, environment exposures, and genetic predisposition (Turner et al., 2023). It affects individuals across all demographics, irrespective of age, sex, race, or baseline health (Bowe et al., 2023; Stephenson et al., 2024). Additionally, vaccination status has been suggested as a potential factor influencing disease trajectory (Pretorius et al., 2021; Byambasuren O et al., 2023; Tran et al., 2023).

To date, more than 13 billion COVID-19 vaccine doses have been administered globally to reduce the impact of the pandemic (WHO, 2024). However, some vaccinated individuals have reported persistent symptoms resembling that seen in acute and Long COVID, leading to the term “vaccine injury” (Scholkmann and May, 2023; Turner et al., 2023). The underlying mechanisms remain poorly understood, with several factors, including vaccine type, host genetics, prior SARS-CoV-2 infection, and timing of the vaccination, potentially influencing any adverse outcomes (Barouch, 2022; Scholkmann and May, 2023; Cruz Cisneros et al., 2024).

COVID-19 vaccines fall into four primary categories: adenovirus vector-based vaccines (e.g., AstraZeneca’s Vaxzevria, Covishield ChAdOx1 and Johnson-Janssen’s Ad26.COV2.S), mRNA-based vaccines (e.g., mRNA, Moderna’s Spikevax mRNA-1273 and Pfizer-BioNTech’s Comirnaty BNT162b2), adjuvanted protein vaccines (e.g., Norvavax’s Nuvaxovid and Covovax NVX CoV 2373), and inactivated virus vaccines (e.g., Sinopharm’s Covilo, Sinovac’s CoronaVac and Bharat Biotech’s Covaxin) (Barouch, 2022). The complex interplay between SARS-CoV-2 infection, immune response, and vaccination presents significant challenges in distinguishing Long COVID from vaccine-related injuries. Misdiagnosis of Long COVID and/or vaccine injury can easily occur due to the overlapping symptoms and the challenge of establishing a clear timeline between infection and/or vaccination-induced issues. This is especially difficult when distinguishing between Long COVID cases caused solely by infection, and cases where vaccination may also involve vaccine injury (**Supplementary Figure 1**). In many cases, timeline of disease onset is undetermined (Scholkmann and May, 2023), making it nearly impossible to delineate causality with confidence.

Current research on Long COVID suggests multiple pathophysiological mechanisms, including autoimmunity, immune dysregulation, viral persistence, gut dysbiosis, and microvascular dysfunction (Davis et al., 2023; Al-Aly and Topol, 2024; Al-Aly et al., 2024; Zollner et al., 2024; Gupta et al., 2025). One of the most noteworthy findings in recent studies is the presence of a hypercoagulable state across COVID-19-related conditions, including Long COVID and vaccine injury (Campello et al., 2022; Nicolai et al., 2023; Shakhidzhanov et al., 2025). The initial SARS-CoV-2 infection induces a pronounced inflammatory response, altering the balance between pro- and anti-coagulant pathways. A persistent heightened inflammatory response can ultimately lead to endothelial dysfunction (Chauhan et al., 2020; Villacampa et al., 2024). In addition, spike protein is capable of inducing fibrinolysis-resistant clotting (Grobbelaar Lize et al., 2021), the severity of which can be influenced by the virulence of the SARS-CoV-2 variant, suggesting that variant-specific virulence may contribute to the development and extent of a hypercoagulable state in post-sequelae conditions (Grobbelaar et al., 2022).

Considering the complex interplay of factors driving Long COVID, it is essential to distinguish its pathological mechanisms from those underlying adverse vaccine side effects. While the timelines of symptom onset and progression may differ between the two, their pathways can overlap, adding to the challenge of disentangling them (**Supplementary Figures S1**, **S2**). Furthermore, it is important to recognise that millions of individuals developed Long COVID prior to the availability of vaccines. Nonetheless, grasping an understanding of these developmental timelines provides valuable insight into the effect of infection versus vaccination, facilitating the subsequent application of a systems approach by both clinicians and researchers (Barouch, 2022; Scholkmann and May, 2023; Turner et al., 2023).

Given the overlapping clinical manifestations of Long COVID and vaccine-related adverse effects, and the inherent difficulty in definitively excluding prior asymptomatic or unrecognised mild SARS-CoV-2 infection, we have defined the disease cohort recruited in this study, as individuals with Post-Vaccination/Post-Infection Syndrome (PV/PIS). This term acknowledges the complexity of distinguishing between vaccine-associated immune dysregulation and post-infectious sequelae, particularly in populations where acute infection timelines may be unclear (asymptomatic or underreported cases). We recognise that both vaccination and SARS-CoV-2 infection (symptomatic or asymptomatic) may contribute to ongoing immune, inflammatory and coagulation abnormalities. By adopting the PV/PIS terminology in this current paper, we aim to avoid premature attribution of causality and instead focus on describing the proteomic and pathophysiological features present in this disease cohort. Proteomic analysis was performed on digested microclot deposits isolated from platelet-poor plasma (PPP) samples to investigate proteins sequestered within these insoluble, pathophysiologically relevant aggregates. This approach enabled us to specifically focus on the proteins trapped within these insoluble microclots, which are increasingly recognised for their potential role in disease pathology (Schofield et al., 2024).

## 2 Materials and methods

### 2.1 Ethical clearance

This research forms a part of a larger study funded by the South African Medical Research Council (SAMRC, grant number 96847), with ethics approval obtained from the Health Research Ethics Council (HREC) at Stellenbosch University, South Africa (project reference number N22/11/133, ID: #26785). The study protocol ensured that all participants were fully briefed on the experimental objectives, potential risks, and all pertinent study details. Additionally, their informed consent was obtained for both sample collection and storage for multi-disciplinary research. Throughout the study and across all research activities, rigorous adherence to ethical standards was meticulously upheld, following the principles outlined in the Declaration of Helsinki, the South African Guidelines for Good Clinical Practice, and the Medical Research Council Ethical Guidelines for Research.

### 2.2 Sample recruitment and patient inclusion criteria

A questionnaire was used to collect information on age, gender, pre-existing co-morbidities, other clinical characteristics, vaccination status, and details regarding the diagnosis of acute COVID, Long COVID, or vaccine-related events that might have resulted in persistent symptom onset. Additionally, data on the condition severity and self-reported symptoms were gathered.

Our study cohort included 16 ostensibly healthy participants who served as the control group (12 females; 4 males; mean age 43.9 ± 15.2). The control group comprised a mix of convalescent individuals who had fully recovered after acute SARS-CoV-2 infection, as well as participants with no reported history of infection; with only one pre-COVID sample included as an ostensibly healthy individual at the time of sample collection. All control participants had received at least one COVID-19 vaccination and/or experienced an acute SARS-CoV-2 infection but did not develop the prolonged symptoms characteristic of PV/PIS or Long COVID, respectively. Participant selection was not based on vaccine type. Among the 16 ostensibly healthy participants, 9 had a history of acute COVID infection, and had fully recovered without prolonged health issues. Two of the healthy controls developed pericarditis after vaccination but recovered fully following early treatment (see **Supplementary Table 1**). The remaining participants did not report a prior infection; however, the possibility of an asymptomatic infection cannot be definitively ruled out. Nucleocapsid (N-protein) antibody testing, which is commonly used to identify prior natural SARS-CoV-2 infection, was not performed on the study cohort, thus prior asymptomatic exposure cannot be excluded (Assis et al., 2021; Jarlhelt et al., 2023; Noble et al., 2025).

The PV/PIS cohort included 14 participants (9 females; 5 males; mean age 59.9 ± 15.8) that met our inclusion criteria. The inclusion criteria for our PV/PIS cohort required participants to have received at least one COVID-19 vaccine between December 2020 and December 2024, with symptoms persisting 12 weeks or longer post-vaccination. By design, all PV/PIS participants were experiencing persistent symptoms for 12 weeks or longer at the time of recruitment, ensuring the cohort represents a chronic disease state. All PV/PIS participants had received only Pfizer-BioNTech vaccines, during the period from mid-2021 to mid-2022. Most of the PV/PIS cohort were vaccinated during the third wave of COVID-19 (May – September 2021, Delta variant peak), shortly before the emergence of the Omicron variant in December 2021 (see **Supplementary Figure 3**). One of the PV/PIS cases presenting with myocarditis shortly after vaccination repeatedly tested negative for SARS-CoV-2 infection (see **Supplementary Table 1**).

For participants with documented acute COVID cases (before or after vaccination), confirmation via PCR or antigen testing was required. Of the 14 PV/PIS participants, 8 had reported prior acute COVID infection, with 50% of the cases occurring pre-vaccination and 50% of the cases occurring post-vaccination (see **Supplementary Table 1**). Exclusion criteria included acute COVID infection at the time of vaccination, or ongoing treatment for acute or Long COVID.

### 2.3 Sample collection

Blood samples were collected via venepuncture by a licensed medical practitioner, or certified phlebotomist. These samples included the following: three 3 mL sodium citrate (3.2%) tubes (BD Vacutainer^®^, 369714), and one 5 mL serum (silicone/polymer gel) blood tube (BD Vacutainer^®^, 367986) for vascular pathology studies. The sodium citrate tubes were then transported to the haematology laboratory at Stellenbosch University. Only one whole blood (WB) sample was centrifuged at 3000 ×g for 15 min at room temperature. The supernatant platelet-poor plasma (PPP) was carefully removed and aliquoted into a 1.5 mL Eppendorf tube and stored at -80 °C until further use. All other samples collected were used as part of research outside the scope of this study.

### 2.4 Fluorescence microscopy

#### 2.4.1 Sample preparation and viewing of platelet poor plasma to study heterogenous amyloid deposits (microclots)

Although not the primary focus of this study, fluorescence microscopy was used to provide a qualitative overview of the extent of abnormal clotting in PV/PIS compared to controls.

At room temperature, within 24h of sample collection, 49 μL of platelet-poor plasma (PPP) was aliquoted into a 1.5 mL Eppendorf tube. The samples were stained with 1 μL of Thioflavin T (ThT, Sigma-Aldrich) with a final concentration of 0.005 mM, while protected from light. The stained samples were incubated in a light-protected container at room temperature for 30 min. After incubation, 3 μL of the stained PPP sample was used to prepare smears for viewing and analysis. This ThT method was previously established to visualise abnormal clotting in various inflammatory conditions (Kell and Pretorius, 2017; Pretorius et al., 2017; Page et al., 2019).

The PPP smears were examined using a Zeiss AxioObserver 7 fluorescent microscope with a Plan-Apochromat 63×/1.4 Oil DIC M27 objective (Carl Zeiss Microscopy, Munich, Germany). For ThT, the excitation wavelength range was set between 450 nm to 488 nm, with an emission range from 499 nm to 529 nm (Pretorius et al., 2020; Venter et al., 2020; Grobbelaar Lize et al., 2021). Representative micrograph images were captured at random, with a minimum of four images acquired per sample.

### 2.5 Proteomics: digestion of heterogenous amyloid deposits (microclots) in platelet poor plasma

#### 2.5.1 Sample preparation and trypsin digestion

For proteomics analysis, 30 platelet-poor plasma (PPP) samples, previously stored at -80 °C, were selected, including controls (n = 16), and PV/PIS (n = 14), and 100 μL of each sample was aliquoted out. These pre-aliquoted samples were then stored at -20 °C until use.

A 20× dilution of the stored PPP was prepared by combining 5 μL of the naïve sample with 45 μL of 10 mM ammonium bicarbonate (NH_4_HC0_3_). The protein concentration of each sample solution was determined using a nanodrop spectrophotometer (ThermoFisher), measuring absorbance at 280 nm.

This sample set had an increased viscosity of the PPP compared to previous studies by Pretorius et al. (2021), and Kruger et al. (2022), necessitating adjustments to the heterogenous amyloid deposits (microclots) analysis protocol outlined by Pretorius et al. (2021). Each sample was standardised using a set volume of 25 μL naïve PPP sample, combined with 100 μL of phosphate-buffered saline (PBS, McKesson) to better mimic physiological conditions. The sample solution was placed in a Falcon tube and rotated on a tube rotator, for 50–60 min, until homogenised. The samples were centrifuged at 12 000 ×g for 10 min at room temperature to separate the heterogenous amyloid deposits (microclots; insoluble fraction of PPP) from the soluble portion. The supernatant was removed, leaving 8 μL of sample solution behind. The remaining solution was reconstituted with 72 μL PBS, to achieve a 10× dilution factor and centrifuged again at 12 000 ×g, for 10 min at room temperature (Pretorius et al., 2021; Kruger et al., 2022).

Carefully, to avoid disturbing the heterogenous microclot pellet, 72 μL of soluble fraction of the sample solution was removed. The pellet was further dissolved by the adding 10 μL of a solution containing 5 mM tris (2-carboxyethyl)phosphine (TCEP, Sigma-Aldrich) and 250 mM triethylammonium bicarbonate (TEAB, ThermoFisher) to the remaining 8 μL of sample solution. The samples were vortexed briefly (5 s), then incubated in a heating block for 30 min at 60 °C, followed by 30 min at 44 °C. After incubation, the samples were briefly centrifuged at room temperature using a mini benchtop centrifuge to re-incorporate any evaporated sample that may have settled in the lid of the Eppendorf tube and then allowed to cool to room temperature.

To further denature the insoluble heterogenous deposits, 20 μL of 50% methanol was added to each sample. To block the cysteine residues, the samples were alkylated with 3 μL of iodoacetamide (protected from light) and incubated at room temperature for 30 min. To each sample, 4 μL of a solution containing dithiothreitol (DTT, Sigma-Aldrich) and TEAB (ThermoFisher) was added. The pellet was further digested by adding 10 μL of a trypsin solution, containing 0.5 μg/μL trypsin (Pierce), and incubated at 37 °C, for 18h.

Following incubation, 5 μL of 5% trifluoroacetic acid (TFA, Sigma-Aldrich), with a final concentration of 1% v/v, was added to each sample to acidify and terminate the reaction. The samples were then centrifuged at 12 000 ×g for 10 min at room temperature to pellet the trypsin precipitate. The supernatant was transferred to conical inserts (ALWSCI technologies). The samples were dried under vacuum for 30 min, using a rotary evaporator. Dried samples were resuspended in 30 μL of analytical grade water, and peptide concentration was measured using a nanodrop spectrophotometer (ThermoFisher), with absorbance measured at 220 nm. After measuring the peptide concentration, the samples were dried again under vacuum using the rotary evaporator. Dried samples were resuspended in 20 μL of a loading buffer containing 2% acetonitrile (Burdick and Jackson) in analytical grade water with 0.1% formic acid (Sigma-Aldrich). The digested heterogenous microclot pellet was loaded directly in the autosampler set to 7 °C.

#### 2.5.2 Liquid chromatography of the digested pellet

Liquid chromatography was conducted as previously described by Pretorius et al. (2021), Kruger et al. (2022), and Nunes et al. (2024), using Thermo Scientific Ultimate 3000 RSLC (ThermoFisher, 2019), equipped with a 20 mm × 100 μm C18 trap column (Thermo Scientific), and a CSH 25 cm × 75 μm with a 1.7 μm particle size C18 column (Waters) analytical column (Pretorius et al., 2021; Kruger et al., 2022; Nunes et al., 2024).

The solvent system for loading consisted of 2% acetonitrile (Burdick and Jackson) with 0.1% formic acid (Sigma-Aldrich) in analytical grade water. Solvent A consisted of analytical grade water, with 0.1% formic acid (Sigma-Aldrich), while solvent B consisted of 100% acetonitrile (Burdick and Jackson) with 0.1% formic acid (Sigma-Aldrich).

Samples were loaded onto the trap column at a flow rate of 2 μL/min, from a temperature-controlled autosampler set 7 °C, using loading solvent. Loading was performed for 4 mins before elution onto the analytical column. The flow rate was set to 300 nL/min, and the gradient was generated as follows: 5% - 30% solvent B over 65 min and 30-45% solvent B from 65–80 min. Chromatography was performed at 45 °C, with the outflow directed to the mass spectrometer using a stainless-steel nano-bore emitter.

#### 2.5.3 Mass spectrometry of the digested pellet

Data independent acquisition (DIA) mass spectrometry analysis was performed using ThermoScientific Fusion mass spectrometer, equipped with a Nanospray Flex ionisation source. The prepared samples were introduced through a stainless-steel nano-bore emitter. Data was collected in a positive mode with spray voltage set 2.0 kV and ion transfer capillary at 290 °C. Polysiloxane ions, at 445.12003 m/z, were used for internal calibration of the spectra.

For MS1 scans, the Orbitrap detector was set to a resolution of 60,000 over a scan range of 375–1500 m/z, with an automatic gain control (AGC) target set to standard. Data acquisition was conducted in profile mode.

In higher-energy C-trap dissociation (HCD) mode, precursor ions were selected for fragmentation using the quadrupole mass analyser with HCD energy set to 30%. The precursor mass range was set to 500–900 m/z, with an isolation window of 20 m/z. Fragment ions were detected using the Orbitrap mass analyser set to a resolution of 30,000. The AGC target was set to custom. Data acquisition was performed in centroid mode.

#### 2.5.4 Mass spectrometry data analysis

The raw data files were processed using FragPipe Analysis Pipeline (version 22.0), with the included DIA SpecLib Quant workflow. The Uniprot human database concatenated with the SARS-CoV-2 database, from UniProt (https://www.uniprot.org/taxonomy/9606; https://www.uniprot.org/uniprotkb?query=694009), was used as reference. MSFragger search parameters included fragment mass tolerances set to 20 parts per million (PPM) for mass calibration and optimisation; and enzyme was set to select trypsin, allowing for 2 mis-cleavages. Fixed modifications were set to carbamidomethyl-C, and variable modifications were set to methionine (M) oxidation, protein end terminal acetylation, and the deamidation of asparagine and glutamine residues (NQ). Peptide spectral match (PSM) validation was done with percolator and rescored using MSBooster in FragPipe. Protein inference was performed using ProteinProphet. Quantification was done using DIA-NN with a false discovery rate (FDR) set to 0.01.

Protein group (pg_matrix.tsv) and experiment annotations (.tsv) files, generated by using the FragPipe Pipeline, were uploaded into FragPipe-Analyst (http://fragpipe-analyst.nesvilab.org/) for statistical analysis and validation. The minimum percentage of non-missing values globally and in at least one condition, was set to 0. The adjusted p-value cutoff was 0.05, and the Log_2_ fold cutoff was 1. Median-centred normalisation was used, with Perseus imputation type and Benjamini-Hochberg method for FDR correction.

Protein-protein interaction networks were viewed using the STRING database (version 12.0, https://string-db.org/). Statistically significant protein changes (determined in the above FragPipe analysis) were uploaded to the multiple protein search tool. Under the clusters tab in STRING, Markov Cluster (MCL) algorithm was selected, and the inflation parameter was set to 3, to perform clustering analysis. Functional enrichment analysis was applied to identify over-represented pathways, molecular functions, and biological processes within each cluster.

Amyloidogenic potential of significant proteins was predicted using AmyloGram (http://biongram.biotech.uni.wroc.pl/AmyloGram/, accessed on 19 March 2025). A cut-off value (probability threshold) of 0.5 was applied. A cut-off value of 0.5 will automatically yield the following values for the following parameters: a sensitivity value set to 0.8658, specificity value set to 0.7852 and a Matthews correlation coefficient (MCC) of 0.6268. These values are automatically adjusted with any changes made to the cut-off value. The amyloid probability and fraction of amyloid residues were recorded for each significant protein change.

### 2.6 Statistical analysis

All data from demographics, including the subsequent statistical evaluations, were processed using GraphPad Prism (version 10.2.3). The Shapiro-Wilk test was used for normality testing. For parametric data, statistical significance was determined using unpaired t-test. A 95% confidence level was applied, with statistical significance considered at p< 0.05. Significance levels are indicated by asterisks (*= p< 0.05; ** = p< 0.01; *** = p< 0.001; and **** = p< 0.0001). Parametric data is presented as mean ± standard deviation (SD). Statistical analysis for proteomics data is detailed above.

### 2.7 Comparative analysis

For contextual comparison, we referenced proteomic findings from a previously published Long COVID cohort analysed under different laboratory conditions (Kruger et al., 2022). This comparator group consisted predominantly of unvaccinated individuals with PCR- or antigen-confirmed SARS-CoV-2 infection that subsequently developed into Long COVID, reflecting a cohort in which infection-related effects could be more readily distinguished from vaccine-related influences.

Comparative analysis was performed using the Spotfire^®^ program (http://spotfire.com/, version 12.0, accessed on 14 April 2025) to visualise and interpret proteomic differences between the current study and previously published Long COVID data by Kruger et al. (2022). Proteins identified as having significant changes in the current study were compared to those reported in the Long COVID dataset (Kruger et al., 2022). To standardise the scale across datasets, fold changes values for downregulated proteins were converted to their reciprocals, such that values <1 reflected downregulation and values >1 reflected upregulation. A scatter plot was generated in Spotfire^®^, with fold change plotted on the x-axis, and p-value on the y-axis. A trend line was included to aid in the visual interpretation of data distribution. Proteins were assessed for overlap and directional consistency between the two studies.

## 3 Results

### 3.1 Study cohort and demographics

Sample demographics are shown in **Table 1** and **Supplementary Figure 2** represents the various ways PV/PIS participants developed their condition. The development and progression of PV/PIS is not exclusive to a single developmental pathway but can manifest through various pathways.

**Table 1:** Sample cohort and demographics from healthy participants, and participants with Post-Vaccination/Post-Infection Syndrome (PV/PIS).

Sample demographics			
Platelet poor plasma stored previously			
Mean age [with ± SD]			
Controls (n=16)		43.9 (±15.2)	
PV/PIS (n=14)		59.9 (±15.8)	
Gender distribution across all groups			
Controls (n=16)		12 Females; 4 Males	
PV/PIS (n=14)		9 Females; 5 Males	
Clinical characteristics of participants			
Variables	% In 16 controls		% In 14 PV/PIS
Chronic medication	12.5		57.1
Smoking			
Previous smoker	0		21.4
Current smoker	0		35.7
Body mass index (BMI)			
Underweight ( 18.5)	0		0
Normal (18.5 24.9)	68.8		50.0
Overweight (25.0 29.9)	12.5		28.6
Obese ( 30.0)	18.8		21.4
Cardiovascular disease	6.3		14.3
Hypertension	6.3		35.7
Arthritis	0		21.4
Cancer	6.3		7.1
Type 2 diabetes	0		21.4
Anaemia	6.3		7.1
Rosacea	6.3		0
Irritable bowel syndrome	6.3		21.4
Hypothyroidism	0		14.3
Dyslipidaemia	25.0		21.4
Anxiety/depression	18.8		50.0
Neurodevelopmental disorders	0		7.1
Percentage (%) of participants vaccinated			
Sample group	Number of participants		Percentage (%)
Controls (n=16)	15 of 16		93.8
PV/PIS (n=14)	14 of 14		100
Type of vaccine administered			
Vaccine type and/or combination	Number of control participants (n = 15)		Number of PV/PIS participants (n = 14)
Unknown vaccine information	1 of 15		0 of 14
Pfizer only	9 of 15		14 of 14
J&J only	4 of 15		0 of 14
Pfizer and J&J	1 of 15		0 of 14

All demographics data was subjected to a Shapiro-Wilks normality test. An unpaired T-test was performed on parametric data, with data are represented as mean ± standard deviation (SD).

The majority of the healthy individuals who reported that they suffered from an acute infection, experienced acute symptoms such as cough (55.6%), sore throat (33.3%), fever (55.6%) and headache (33.3%) (**Supplementary Table 1**). Similarly, among the 14 PV/PIS participants, 8 had a prior acute COVID infection, with most reporting cough (62.5%), sore throat (62.5%), fever (87.5%), myalgia (62.5%), malaise (50.0%) and dysgeusia (37.5%) (**Supplementary Table 1**). These symptoms resolved soon after the infection cleared, with no persistent symptoms reported.

Regarding vaccination status, 93.8% of the healthy individuals were vaccinated, receiving either Pfizer, J&J, or a combination of both. In contrast, all PV/PIS participants had exclusively received Pfizer (**Table 1**). Notably, participant selection for this study was not based on vaccination type.

### 3.2 Heterogenous amyloid deposits (microclots) in platelet-poor plasma as viewed with fluorescence microscopy

**Figure 1:**
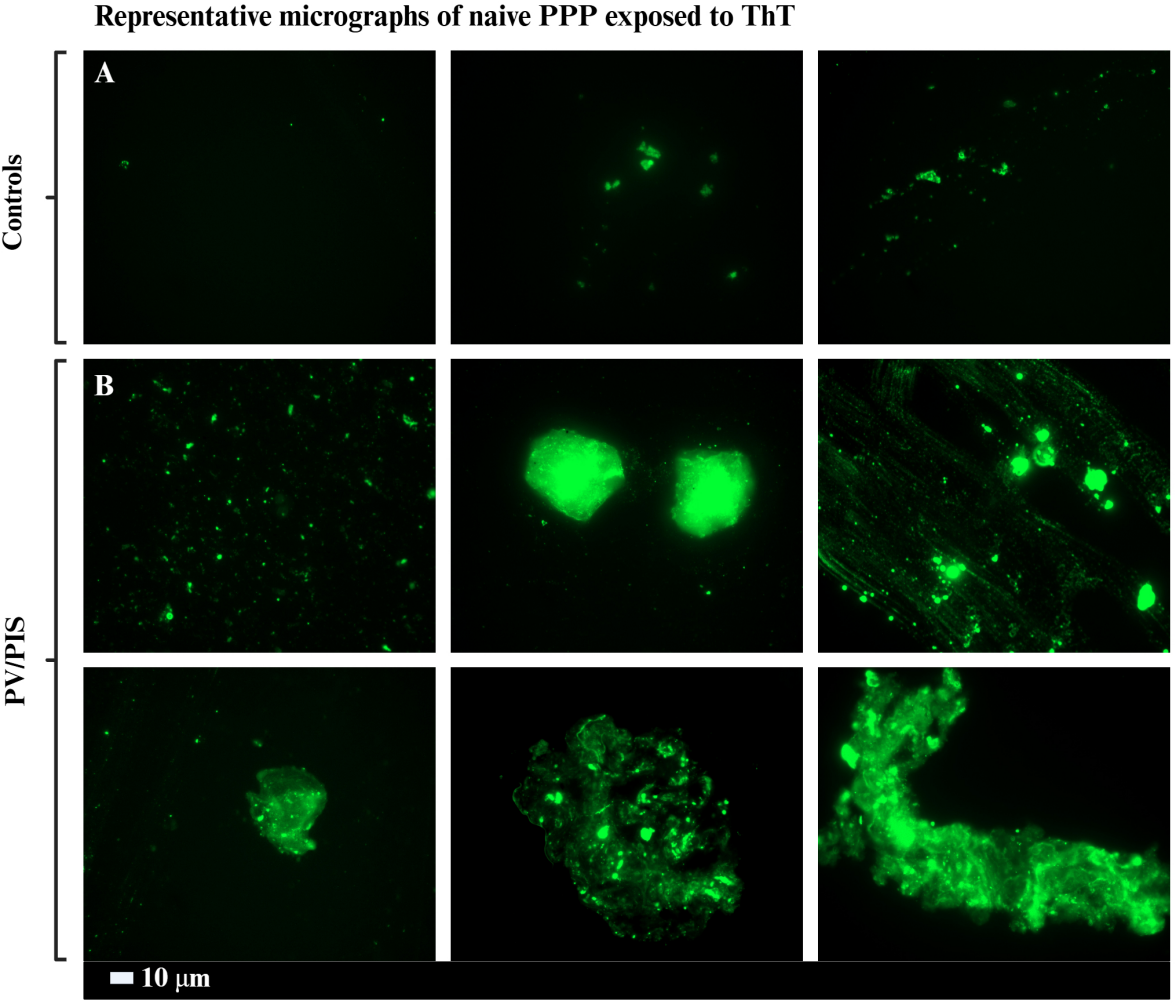
Representative static fluorescent micrographs of platelet poor plasma (PPP) exposed to thioflavin T (ThT). **(A)** shows representative heterogenous amyloid deposits (microclots) in healthy (control) individuals. **(B)** shows extensive formation of heterogenous amyloid deposits (microclots) in PV/PIS participants.

Previous studies (Pretorius et al., 2020; Pretorius et al., 2021) have shown that naïve PPP from healthy individuals and type 2 diabetes mellitus (T2DM) participants exhibit significantly fewer heterogenous amyloid deposits (microclots) upon exposure to ThT compared to acute COVID and Long COVID participants. In this study, we demonstrate that PV/PIS participants also exhibit substantial formation of heterogenous amyloid deposits (microclots), comparable to that seen in other Long COVID studies (Altmann et al., 2023; Sideratou and Papaneophytou, 2024). **Figure 1** provides examples of naïve PPP samples exposed to ThT from both control and PV/PIS groups.

### 3.3 Liquid chromatography-mass spectrometry (LC-MS) of digested microclot pellet deposits in platelet-poor plasma

**Figure 2:**
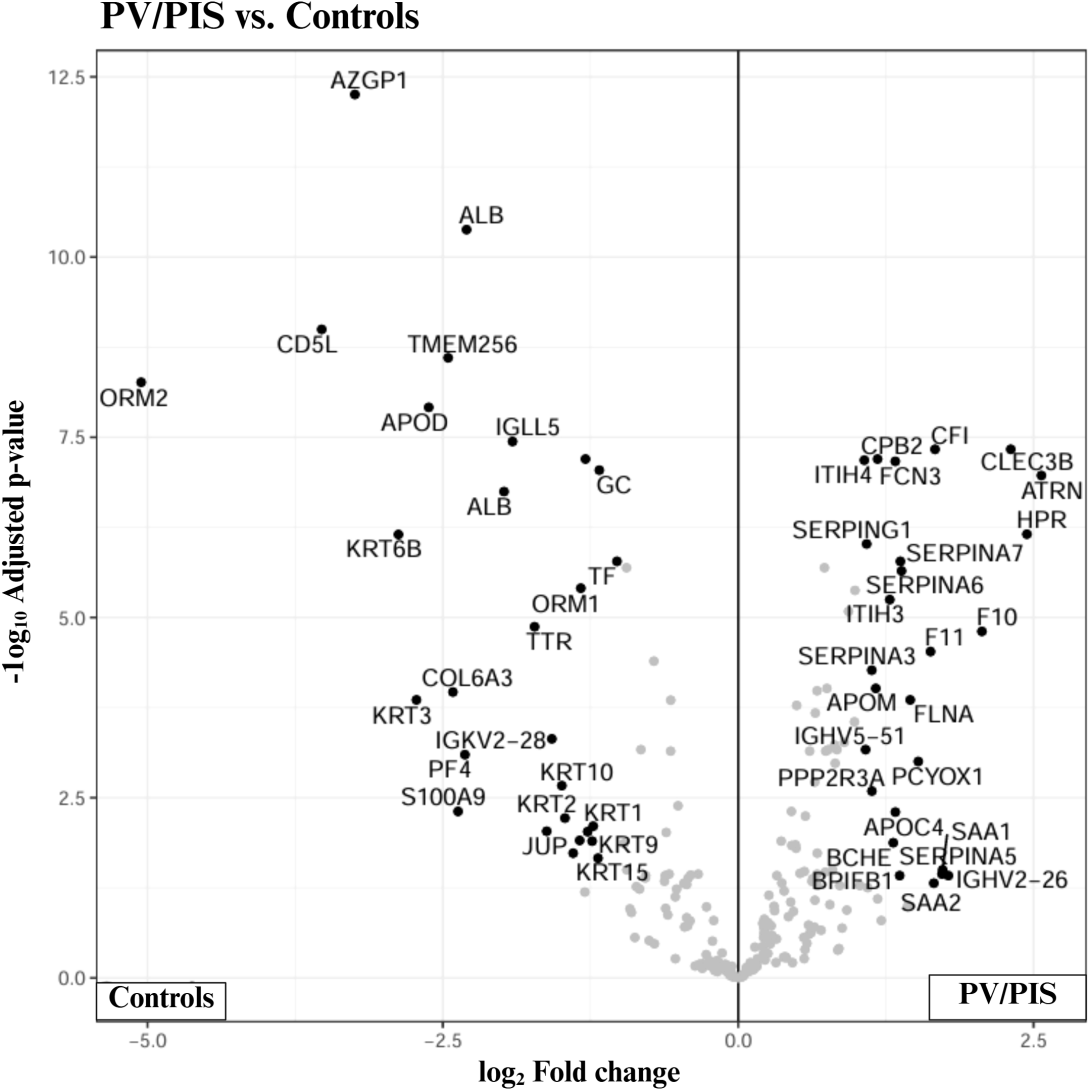
Volcano plot illustrating the distribution of proteins for pairwise comparison (controls vs. PV/PIS). The plot highlights significant proteins, represented by black dots, giving a preliminary overview of protein changes between sample groups. The x-axis denotes fold change to indicate the degree of up or downregulation in PV/PIS relative to controls, with negative values indicating downregulation and positive values indicating upregulation. The y-axis represents the -log10 of p-values, reflecting the statistical significance of the observed changes, with significance taken as p<0.05. Statistical analysis was done using FragPipe Analyst (http://fragpipe-analyst.nesvilab.org/).

**Figure 3:**
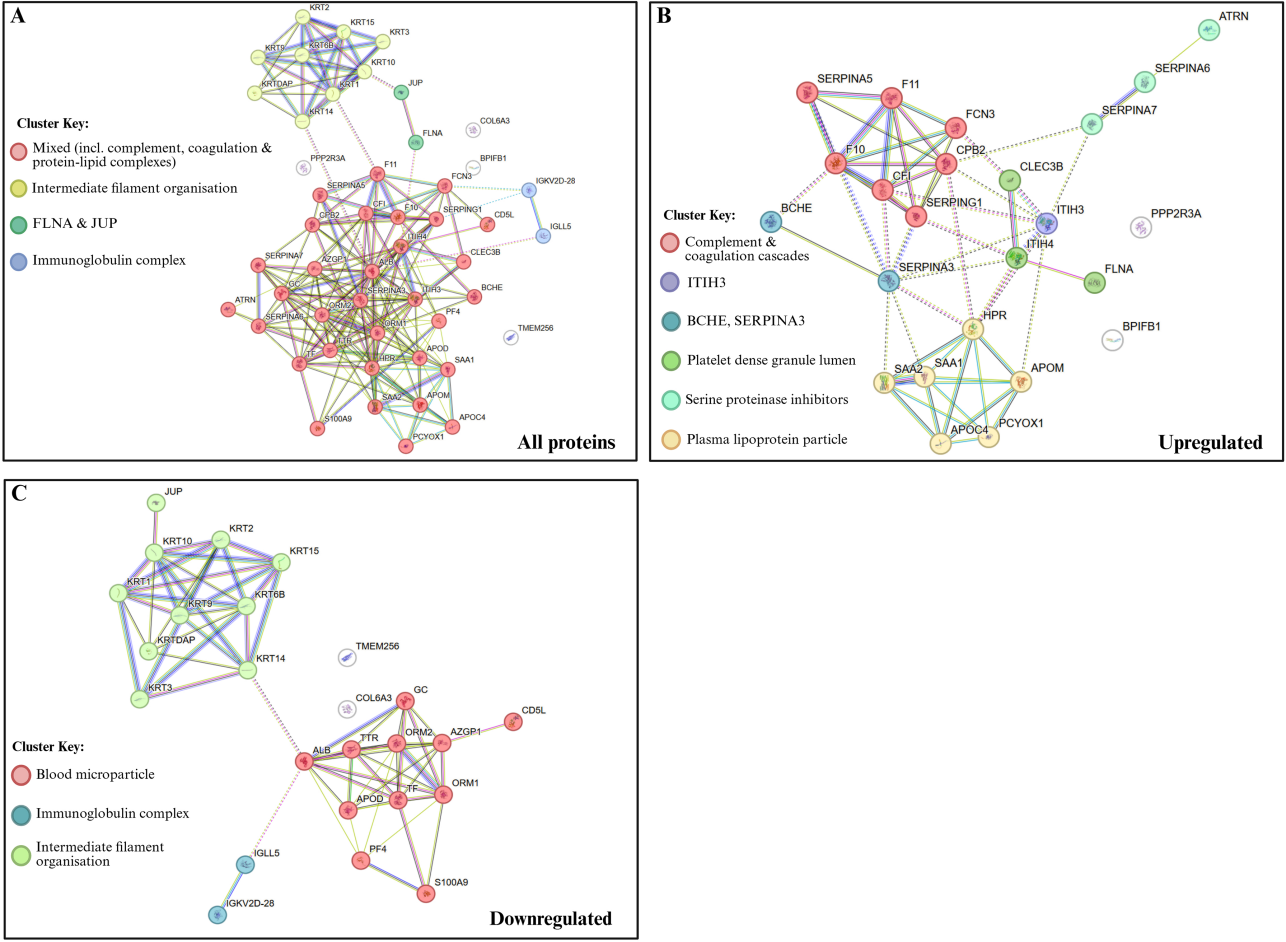
Functional protein-protein interaction networks and clustering analysis for the pairwise comparison of PV/PIS vs. controls. **(A)** functional protein interaction network of all significant differentially expressed proteins. **(B)** network representation of proteins significantly upregulated in PV/PIS samples compared to controls. **(C)** network representation of proteins significantly downregulated in PV/PIS samples compared to controls. Nodes represent proteins, and edges (solid and dotted lines) indicate predicted functional associations. Solid lines indicate strong-high confidence interactions and/or direct physical interactions; however, dotted lines indicate weaker or indirect interactions, functional associations or computationally predicted relationships. Nodes of the same colour representing functionally related groups of proteins, while white nodes are additional interactors suggested by STRING. Clustering and visualisation were performed using the STRING database (version 12.0, https://string-db.org/).

Proteomics data analysis of control (n = 16) and PV/PIS (n = 14) samples was performed using FragPipe Analysis Pipeline (version 22.0) and FragPipe-Analyst (http://fragpipe-analyst.nesvilab.org/). Significant protein results are shown below in **Table 2**. This study identified 26 proteins that were significantly downregulated and 26 proteins that were significantly upregulated in PV/PIS group compared to controls. Changes in protein levels are represented by fold change, with downregulated proteins denoted as reciprocal values. **Figure 2** shows a volcano plot for an overview of protein distribution for the pairwise comparison (controls vs. PV/PIS). **Figure 3** shows an overview of functional protein clusters for proteins that are up- or downregulated by more than 1-fold with a p-value< 0.05. Functional protein cluster changes in PV/PIS can give systematic indications of pathways up or downregulated in PV/PIS.

**Table 2:** Significant protein changes in pairwise analysis of heterogenous amyloid deposits (microclots) from PV/PIS individuals and controls.

Downregulated proteins in PV/PIS compared to controls			
Protein name	Protein ID	Fold change	*P* value
Alpha-1-acid glycoprotein 2	P19652	0.198	<0.001
CD5 antigen-like	O43866	0.283	<0.001
Zinc-alpha-2-glycoprotein	P25311	0.309	<0.001
Keratin, type II cytoskeletal 6B	P04259	0.347	<0.001
Keratin, type II cytoskeletal 3	P12035	0.368	<0.001
Apolipoprotein D	P05090	0.382	<0.001
Transmembrane protein 256	Q8N2U0	0.407	<0.001
Collagen alpha-3 chain	P12111	0.415	<0.001
Protein S100-A9	P06702	0.422	0.001
Platelet factor 4	P02776	0.433	<0.001
Albumin	P02768	0.435	<0.001
Immunoglobulin lambda-like polypeptide 5	B9A064	0.524	<0.001
Transthyretin	P02766	0.581	<0.001
Keratinocyte differentiation-associated protein	P60985	0.617	0.003
Immunoglobulin kappa variable 2-28	A0A075B6P5	0.633	<0.001
Keratin, type I cytoskeletal 10	P13645	0.671	<0.001
Keratin, type II cytoskeletal 2 epidermal	P35908	0.680	0.002
Junction plakoglobin	P14923	0.746	0.004
Alpha-1-acid glycoprotein 1	P02763	0.752	<0.001
Immunoglobulin kappa light chain	P0DOX7	0.775	<0.001
Keratin, type I cytoskeletal 14	P02533	0.787	0.003
Keratin, type I cytoskeletal 9	P35527	0.806	0.004
Keratin, type II cytoskeletal 1	P04264	0.813	0.002
Keratin, type I cytoskeletal 15	P19012	0.840	0.008
Vitamin D-binding protein	P02774	0.847	<0.001
Serotransferrin	P02787	0.971	<0.001
Upregulated proteins in PV/PIS compared to controls			
Protein name	Protein ID	Fold change	*P* value
Inter-alpha-trypsin inhibitor heavy chain H4	Q14624	1.07	<0.001
Immunoglobulin heavy variable 5-51	A0A0C4DH38	1.08	<0.001
Plasma protease C1 inhibitor	P05155	1.09	<0.001
Alpha-1-antichymotrypsin	P01011	1.13	<0.001
Serine/threonine-protein phosphatase 2A regulatory subunit B	Q06190	1.13	<0.001
Apolipoprotein M	O95445	1.16	<0.001
Carboxypeptidase B2	Q96IY4	1.18	<0.001
Inter-alpha-trypsin inhibitor heavy chain H3	Q06033	1.28	<0.001
Cholinesterase	P06276	1.31	0.004
Ficolin-3	O75636	1.33	<0.001
Apolipoprotein C-IV	P55056	1.33	0.001
Thyroxine-binding globulin	P05543	1.37	<0.001
BPI fold-containing family B member 1	Q8TDL5	1.37	0.016
Corticosteroid-binding globulin	P08185	1.38	<0.001
Filamin-A	P21333	1.46	<0.001
Prenylcysteine oxidase 1	Q9UHG3	1.52	<0.001
Coagulation factor XI	P03951	1.63	<0.001
Serum amyloid A-2 protein	P0DJI9	1.66	0.022
Complement factor I	P05156	1.67	<0.001
Serum amyloid A-1 protein	P0DJI8	1.72	0.014
Plasma serine protease inhibitor	P05154	1.73	0.012
Immunoglobulin heavy variable 2-26	A0A0B4J1V2	1.78	0.015
Coagulation factor X	P00742	2.06	<0.001
Tetranectin	P05452	2.31	<0.001
Haptoglobin-related protein	P00739	2.44	<0.001
Attractin	O75882	2.57	<0.001

P-values smaller than 0.001 are indicated as <0.001, while values greater than this are reported to three significant decimal places. Downregulated proteins are represented using reciprocal values.

After sample homogenisation and trypsinisation, this mass spectrometry-based proteomics analysis revealed an increase in coagulation proteins, including factors X and XI, as well as an increase in acute inflammatory response proteins, including serum amyloid A1 (SAA1) and serum amyloid A2 (SAA2). In contrast, proteins associated with immune response, such as Protein S100A9 and other immunoglobulin complex components, were downregulated in PV/PIS participants compared to controls.

### 3.4 Comparative overview of PV/PIS cohort vs. a previously described Long COVID cohort

**Figure 4:**
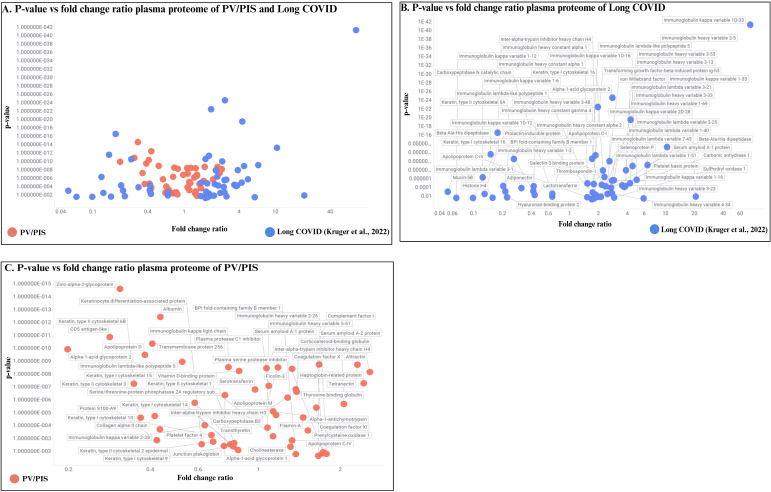
Comparative visualisation of proteomic profiles in PV/PIS participants and a previously published Long COVID cohort (Kruger et al., 2022). **(A)** all proteins found to be significantly expressed in both the current PV/PIS cohort and the Long COVID cohort (Kruger et al., 2022). **(B)** proteins significantly expressed in the Long COVID (Kruger et al., 2022). **(C)** proteins significantly expressed in the PV/PIS cohort as identified in this study. Comparative analysis was performed using Spotfire^®^ program (http://spotfire.com/, version 12.0, accessed on 14 April 2025).

Using the Spotfire^®^ program (http://spotfire.com/, version 12.0, accessed on 14 April 2025), plasma proteomic changes in the PV/PIS cohort were visualised in a scatter plot alongside the proteins reported in a previously published Long COVID proteomic study by Kruger et al. (2022). In **Figures 4A**, a broader distribution of protein changes is observed in the Long COVID group (blue dots), while PV/PIS cohort displays a tighter pattern of protein changes (red dots). **Figures 4B, C**, allow for visual identification of the different proteins significantly up- or downregulated in Long COVID and PV/PIS, respectively.

## 4 Discussion

Since the onset of the COVID-19 pandemic, several SARS-CoV-2 vaccines have been developed to reduce transmission rates and enhance immunity (Rotshild et al., 2021; Eyre David et al., 2022; Marcelin et al., 2022; Echaide et al., 2023). Among these, mRNA-based vaccines, such as Pfizer-BioNTech (BNT162b2) and Moderna (mRNA-1273), were widely deployed due to their rapid availability and their efficacy across different populations; but spike protein mutations have contributed to immune evasion and reduced vaccine efficacy, potentially influencing downstream immunopathological responses (Grobbelaar et al., 2022; Schofield et al., 2024; Sideratou and Papaneophytou, 2024). Most PV/PIS participants were vaccinated shortly before the emergence of Omicron variants (see **Supplementary Figure 3**), suggesting that both vaccine-induced spike protein exposure and residual immune memory may interact with predisposing host factors to shape proteomic outcomes. Although our control cohort included mixed vaccine types, the uniform mRNA vaccination among PV/PIS participants allow for contextual characterisation of cohort-specific proteomic changes, while acknowledging variability in controls as a limitation.

As a pilot study, our aim was to adopt a broad overview to identify untargeted proteomic differences in PV/PIS compared to controls. In this discussion we focus primarily on coagulation factors, acute phase reactants (APRs), and inter-alpha trypsin inhibitor (ITI) family proteins. While individual proteins within these groups often showed modest fold-changes, we highlight them, as subtle alterations in protein classes can collectively contribute to the recurring triad of hypercoagulation, inflammation, and immune dysregulation (Davis et al., 2021; Proal and VanElzakker, 2021; Sekulovski et al., 2023) observed in both our PV/PIS cohort and previous Long COVID cohorts. We note the importance of protein isoforms and their amyloidogenic potential in driving thrombo-inflammatory pathophysiology (Ahn et al., 2017; Rehiman et al., 2022; Kell and Pretorius, 2024) in both conditions.

### 4.1 Inflammation, immune dysregulation and amyloidogenic proteins in PV/PIS

Proteomic profiling revealed significant changes in APRs, immune modulators, and coagulation-related factors in PV/PIS participants relative to controls. Elevated positive APRs, including serum amyloid A (SAA1: 1.72-fold; SAA2: 1.66-fold) and attractin (ATRN: 2.57-fold), alongside decreased albumin (0.435-fold), indicate a sustained inflammatory environment in PV/PIS participants (**Table 2**). These APRs are non-specific markers of inflammation, but their persistent upregulation can influence immune cell recruitment and cytokine signalling (Mohanty et al., 2025), particularly within Th17-skewed pathways (Kell and Kenny, 2016; Lee et al., 2020; Sack, 2020; Barberis et al., 2021; Jayaram et al., 2021; Martonik et al., 2021; Supriya et al., 2021; Akuzum and Lee, 2022; Echaide et al., 2022) (**Supplementary Figure 5**). SAA proteins are known to activate monocytes and dendritic cells, potentially triggering the release of damage- and pathogen-associated molecular pattern molecules (DAMPs and PAMPs), thereby amplifying innate immune responses (Cicchinelli et al., 2024). The concurrent reduction in albumin is consistent with amino acid redistribution to support enhanced synthesis of positive APRs (Gruys et al., 2005; Ahmed et al., 2012).

Amyloidogenic potential, assessed via the AmyloGram predictive computational model, revealed high misfolding propensity for SAA1 (score 0.793) and SAA2 (score 0.845), despite a small fraction of amyloidogenic residues (0.230 and 0221, respectively; see **Supplementary Table 2**). ATRN similarly exhibited a strong amyloidogenic potential profile (score 0.918). Persistent inflammatory states, characterised by chronic upregulation of APRs, are known to create permissive conditions for protein misfolding (Sack, 2018; Sack, 2020; Kim et al., 2022). While the capacity of SAA isoforms or ATRN to form amyloid fibrils in PV/PIS has yet to be experimentally validated, the proteomic profiles identified here point to a sustained pro-inflammatory environment that could facilitate such misfolding events. This raises the possibility that aberrant protein dynamics contribute to vascular dysfunction and the persistence of symptoms in PV/PIS individuals.

Chronic pro-inflammatory environments have been shown to drive downstream pathophysiological clotting and vascular injury (Wang et al., 2012). In line with this, PV/PIS participants exhibited changes in coagulation-related proteins, with the upregulation of intrinsic coagulation factors X (2.06-fold) and factor XI (1.63-fold), and the downregulation of platelet factor 4 (PF4; 0.433-fold; **Table 2**). Reduced PF4 has been associated with enhanced IL-17 production and Th17-mediated inflammation, linking coagulation changes to immune dysregulation (Chakraverty, 2014; Shi et al., 2014). Although the observed upregulation of factor XI was modest (<2-fold), it remains noteworthy as part of a broader pattern of coagulation protein alterations. Future validation studies should consider coagulation-related proteins as a collective network, including pro-coagulants factors such as VWF, α2-antiplasmin, and others, to comprehensively characterise thrombo-immune dysregulation in PV/PIS.

### 4.2 Contextually comparing PV/PIS with Long COVID

Comparative analysis with Long COVID proteomic datasets (Kruger et al., 2022) revealed distinct molecular profiles between Long COVID and PV/PIS. Proteins significantly up- or downregulated in PV/PIS largely did not overlap with Long COVID participants (**Figure 4A**, **Supplementary Figure 6**). The divergent profiles, particularly in the inter-alpha-trypsin inhibitor (ITI) family, suggesting that while both conditions share clinical manifestations, the underlying molecular drivers differ. This warrants future investigations into disease-specific biomarker panels.

Within the ITI family, ITIH3 (1.28-fold) and ITIH4 (1.07-fold) were slightly elevated in PV/PIS participants. These proteins are known to contribute to the maintenance of extracellular matrix integrity and modulation of immune signalling (Jiang et al., 2019; Zhang et al., 2024), with ITIH4 previously characterised as an APR (Hamm et al., 2008; Pihl et al., 2021). Computational predictions using AmyloGram indicated high amyloidogenic potential for ITIH3 (0.913) and ITIH4 (0.902; **Supplementary Table 2**), suggesting that these proteins could participate in cross-seeding with other amyloidogenic proteins, such as fibrinogen. Although amyloidogenic cross-seeding has been primarily studied in neurodegenerative diseases (Anand et al., 2018; Chaudhuri et al., 2019; Ke et al., 2020), this mechanism may also contribute to chronic inflammatory conditions such as PV/PIS, where multiple proteins that are upregulated, including members of the ITI family, possess a high potential to misfold under pathological conditions. Even the modestly significant fold changes, as observed in ITIH3 and ITIH4 in our study, could initiate a “snowball effect” facilitating amyloidogenic cross-seeding and disproportionate incorporation into insoluble heterogeneous amyloid deposits (microclots), thereby amplifying their pathological impact beyond what is suggested by fold change alone. This hypothesis, however, requires experimental validation in post-COVID and post-vaccination cohorts to confirm its relevance. Furthermore, future studies should consider investigating the association between amyloidogenic ITI family proteins and coagulation-related proteins to better understand the hypercoagulable phenotype observed in both Long COVID and PV/PIS.

Although the fold-change differences were modest (<2-fold), these alterations are notable when compared to previously reported Long COVID cohorts, where ITIH1 and ITIH2 upregulation predominated (Kruger et al., 2022). This pattern illustrates the concept of “symptomatic overlap with mechanistic heterogeneity” in Long COVID and PV/PIS, highlighting that similar clinical presentations may arise from distinct molecular pathways. The observed ITIH3/ITIH4 profile represents a preliminary, isoform-specific distinction between PV/PIS and Long COVID, providing a rationale for future targeted proteomic studies. Investigating protein families with multiple isoforms may reveal whether subtle differences in isoform expression are unique to PV/PIS, Long COVID, or other inflammatory conditions, and whether these differences influence disease outcomes. It remains to be experimentally established whether initial triggers in Long COVID and PV/PIS (infection versus vaccination) influence which isoforms are mechanistically relevant in the onset and progression of these conditions.

### 4.3 Implications and future directions

This pilot study provides a broad proteomic overview of PV/PIS, highlighting protein-level changes linked to persistent inflammation, coagulopathy and contributing to immune dysregulation. By considering fold-change, amyloidogenic potential, and immune relevance, we identify candidate proteins for further validation and potential biomarker development in PV/PIS. Importantly, PV/PIS shows mechanistic divergence from Long COVID, underscoring that shared symptoms do not equate to identical pathophysiology. Future research should test correlations with Th17 markers and pro-coagulant factors, assess the amyloidogenic potential of key immune modulatory and coagulation proteins, and evaluate isoform-specific changes in targeted proteomic analyses of PV/PIS and Long COVID.

### 4.4 Study limitations

Integrating proteomic profiles with patient-reported outcomes will be essential to contextualising PV/PIS, as variability in genetic predisposition, epigenetic factors and other clinical characteristics can contribute divergent disease trajectories. Multimodal analyses could support individualised management and targeted interventions, effectively bridging biomarker discovery with clinical application. Notably, in the context of this study, two controls developed pericarditis post-vaccination, which resolved with early treatment. While limited in number, these cases highlight the potential genetic or immunological predispositions to rare-vaccine-related events (Hamm et al., 2008; Zhang et al., 2024) and underscores the importance of including phenotypically “healthy” controls to reflect the broader, “real-world” population, in which most individuals have variability in their baseline health.

More broadly, the small cohort size of this study, limits generalisability and emphasises the need for validation in larger, longitudinal studies. Additionally, the PV/PIS cohort was older than the control group (mean 59.9 ± 15.8 vs. 43.9 ± 15.2, respectively), which could contribute to differences in baseline inflammation, immune function, and coagulation, representing a potential confounding factor.

Furthermore, the control cohort included mixed vaccine types and recovery histories; while sub-analyses by vaccine type could be informative, the small sample size in this pilot study precludes robust stratified comparisons. Additionally, nucleocapsid (N-protein) antibody testing was not performed, which limits the ability to definitively exclude prior asymptomatic SARS-CoV-2 infection in both control and PV/PIS participants (Assis et al., 2021).

Finally, given the pilot nature of this study, the proteomic differences observed should be interpreted as preliminary and associative, rather than causative. Future work should consider vaccine types and balanced sex distribution among both control and PV/PIS cohorts.

## 5 Conclusion

PV/PIS is characterised by sustained upregulation of APRs, immune modulators, and coagulation factors. While some of these alterations overlap with Long COVID, our pilot study suggests that protein isoforms may provide molecular distinctions between the two conditions. Importantly this work established a preliminary proteomic framework that can guide the identification of candidate biomarkers for clinical differentiation of PV/PIS from Long COVID. By defining these molecular signatures, our study lays the groundwork for the development of targeted diagnostic assays and ultimately personalised therapeutic strategies, highlighting the translational potential of proteomics in understanding and managing vaccine-related syndromes.

## Data Availability

The datasets generated and analysed during the current study are securely stored in the institutional data repository and are available from the corresponding author upon reasonable request..
