# Supplementary material for: Proteomic signatures of COVID-19 Post-Vaccination/Post-Infection Syndrome (PV/PIS): insights into immune dysregulation and coagulopathy
Source: Front Cell Infect Microbiol. 2026 Mar 25;16:1753348. doi: 10.3389/fcimb.2026.1753348 (PMC13056885; doi:10.3389/fcimb.2026.1753348)
Supplement: Supplementary file 1 [file DataSheet1.pdf]

Supplementary Material

1 Understanding the cohort: Additional demographics

1.1 Diagnostic ambiguity between Long COVID and vaccine-related injuries

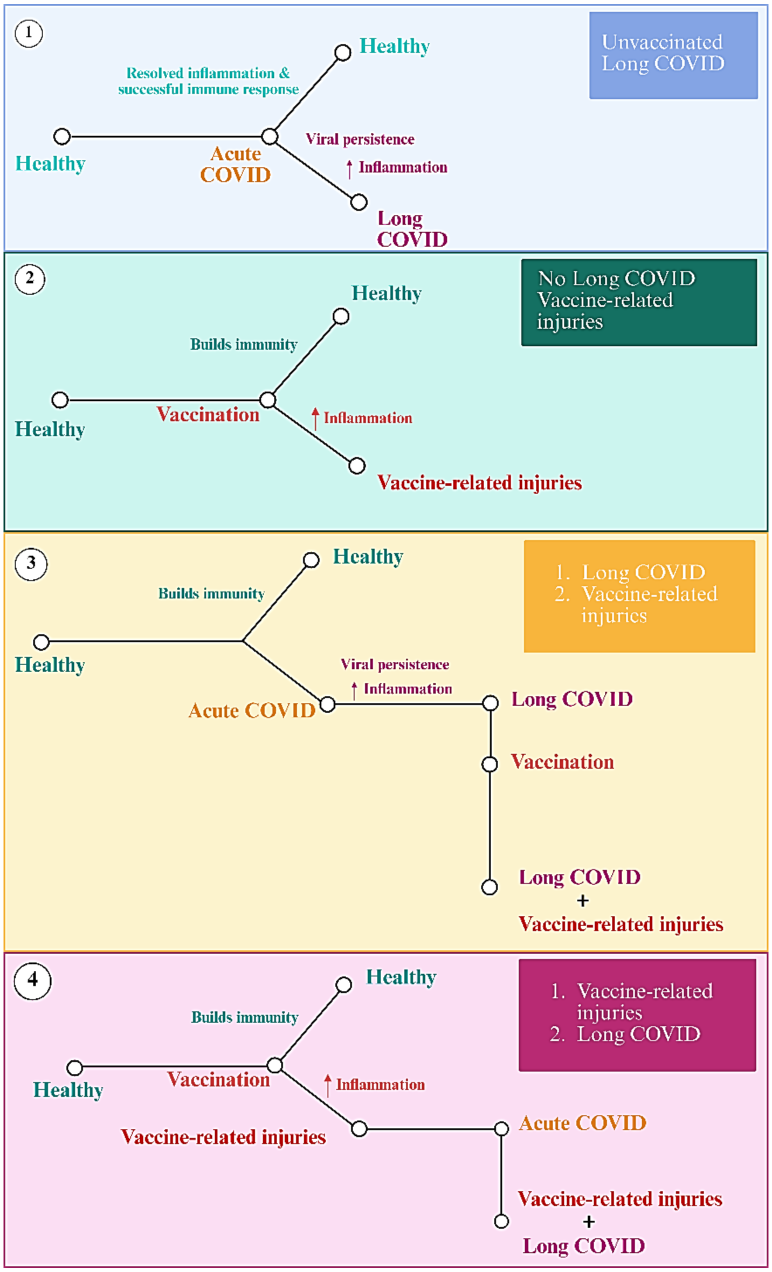

Supplementary Figure 1. Establishing the connection between Long COVID and vaccine-related injuries. The development of Long COVID and vaccine injury can follow various pathways. 1) a healthy unvaccinated individual may contract acute COVID, subsequently recovering to a healthy state; or experience worsening symptoms, which can lead to the development of "pure Long

COVID". 2) a healthy individual who receives a vaccination may either establish immunity and remain ostensibly healthy or may encounter vaccine-related injuries without experiencing acute COVID infection. 3) a previously healthy individual may initially contract acute COVID that progresses into Long COVID. If this Long COVID patient becomes vaccinated, it could potentially exacerbate their condition, resulting in a dual diagnosis of both Long COVID and vaccine injury. 4) a previously healthy individual gets vaccinated and may develop vaccine-related injuries. Subsequently, the patient contracts acute COVID, which progresses into Long COVID, leading to a dual diagnosis of vaccine injury followed by Long COVID. This highlights the intricate interplay and complexity inherent to these conditions. Created in https://Biorender.com.

1.2 Disease developmental pathways of the study cohort

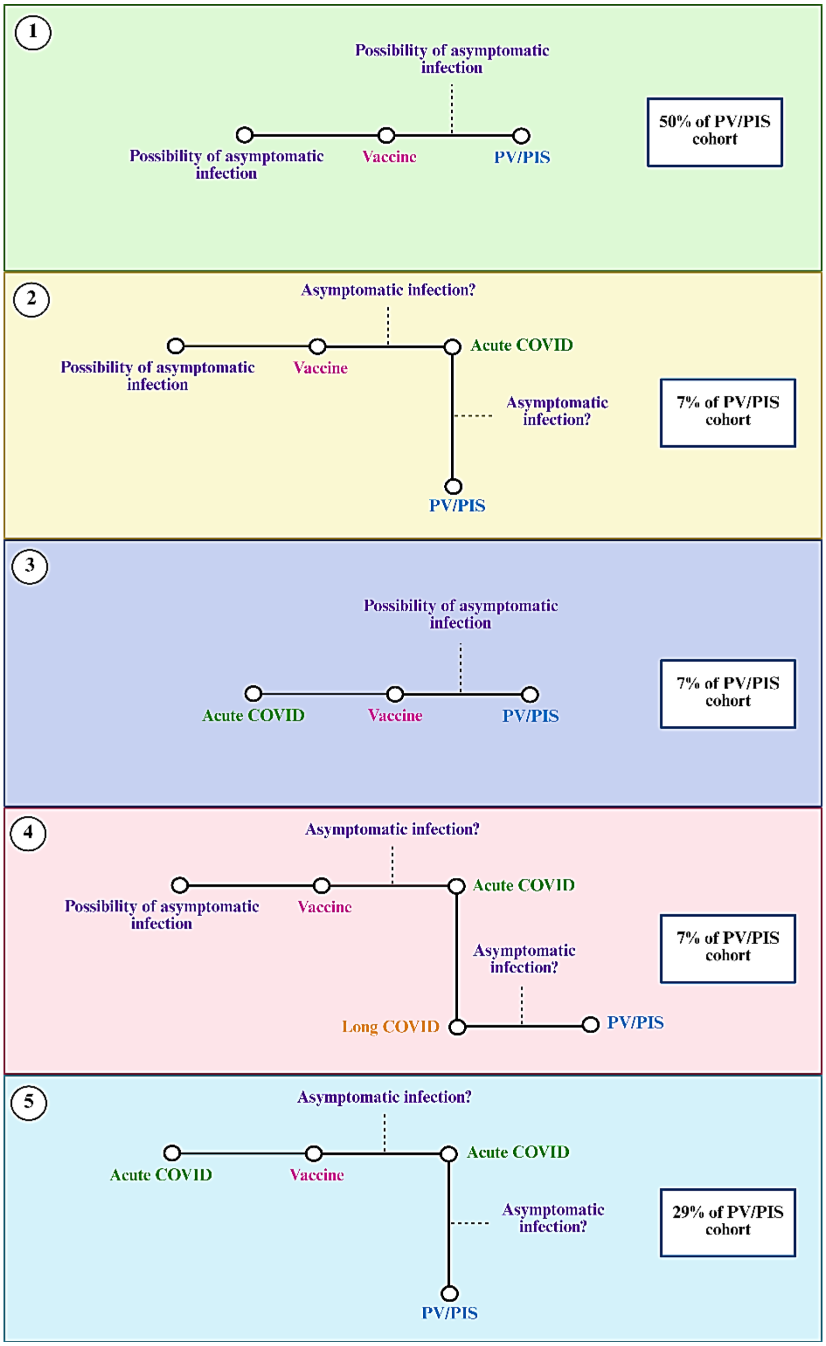

Supplementary Figure 2. Developmental pathways leading to Post-Vaccination/Post-Infection (PV/PIS). 1) shows 50% of PV/PIS cases were directly caused by the vaccination itself, representing pure post-vaccination complications. 2) shows that 7% of PV/PIS cases developed acute COVID following vaccination, exacerbating the vaccine's effects. 3) shows 7% of PV/PIS cases had prior acute COVID infection before vaccination. 4) shows that 7% of PV/PIS cases were vaccinated before contracting acute COVID, which later progressed to Long COVID, intensifying the vaccine's impact, resulting in post-vaccination complications and Long COVID. 5) shows that 29% of PV/PIS cases were vaccinated between acute COVID infections, further exacerbating the vaccine's effects. It is important to note that possible asymptomatic exposure was not accounted for in this figure. Created in https://Biorender.com.

1.3 SARS-CoV-2 variant peak and participant vaccine administration

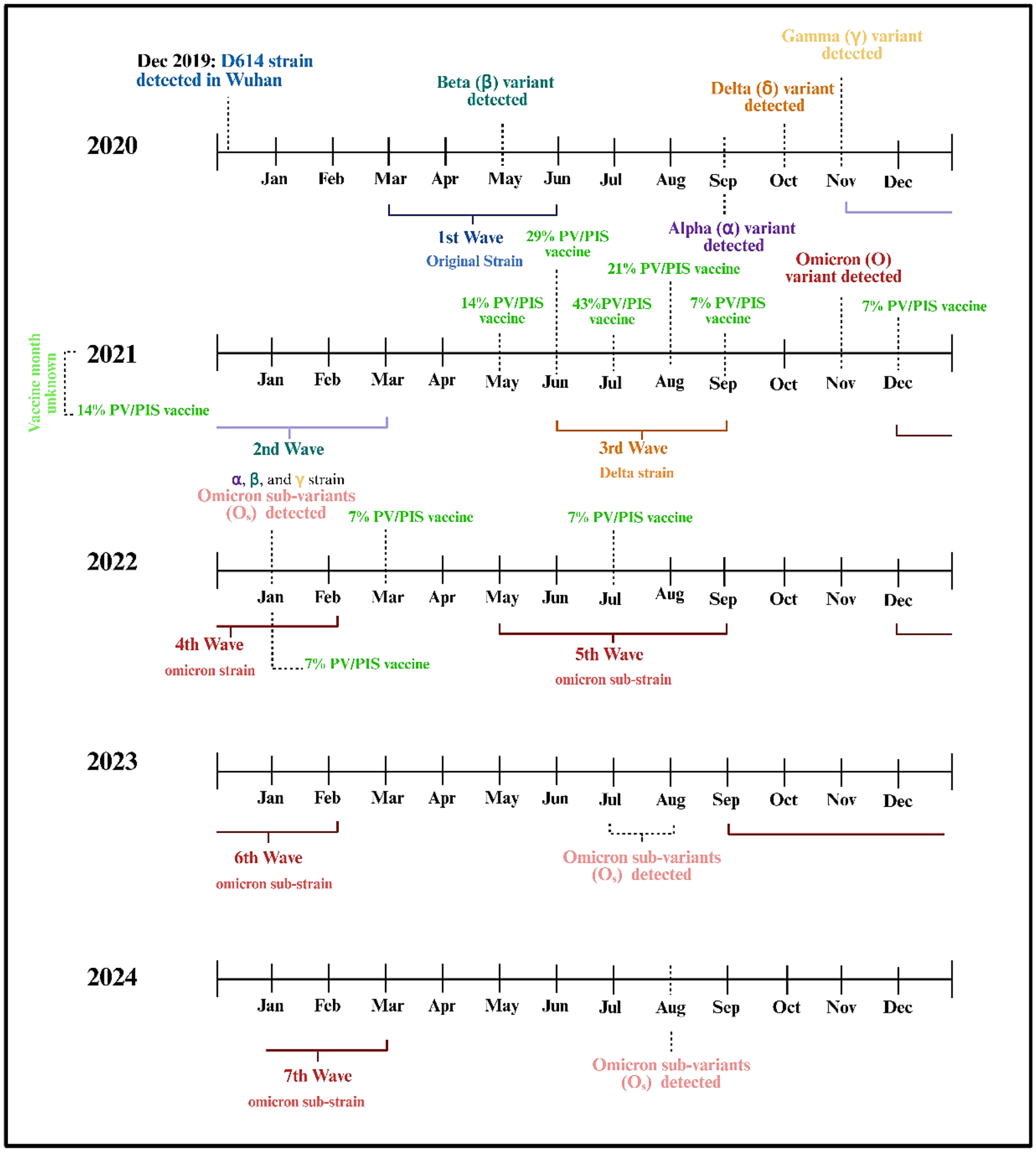

Supplementary Figure 3. Timeline of SARS-CoV-2 variant detection and vaccine administration for the Post-Vaccination/Post-Infection Syndrome (PV/PIS) cohort. The majority individuals in our

PV/PIS cohort received their vaccination during the third wave of COVID-19 (at the peak of Delta variant) in 2021, prior to the first detection of Omicron variant. All PV/PIS participants were vaccinated with mRNA vaccines (Pfizer-BioNtech), which may explain the decrease in efficacy observed against the more recently emerged SARS-CoV-2 variants. Created in https://Biorender.com.

1.4 Additional participant demographics

Supplementary Table 1. Demographics of participants in both control and PV/PIS groups who experienced acute COVID, including the number of cases that developed Long COVID. This table highlights the complexity of distinguishing between Long COVID and PV/PIS.

Sample demographics

Platelet poor plasma stored previously

Percentage (%) of participants vaccinated

**Sample group**	**Number of participants**	**Percentage (%)**
Controls (n=16)	15 of 16	93.8
PV/PIS (n=14)	14 of 14	100

Type of vaccine administered

**Vaccine type and/or combination**	**Number of control participants (n = 15)**	**Number of PV/PIS participants (n = 14)**
Unknown vaccine information	1 of 15	0 of 14
Pfizer only	9 of 15	14 of 14
J&J only	4 of 15	0 of 14
Pfizer and J&J	1 of 15	0 of 14

Number of vaccinations

**Number of vaccine doses received**	**Number of control participants (n = 15)**	**Number of PV/PIS participants (n = 14)**
Unknown vaccine information	3 of 15	0 of 14
1 dose	4 of 15	4 of 14
2 doses	3 of 15	8 of 14
>2 doses	5 of 15	2 of 14

Symptoms experienced after vaccination

**Symptom**	**Number of control participants (n = 15)**	**Number of PV/PIS participants (n = 14)**
Fever	3 of 15	2 of 14
Chills	2 of 15	0 of 14
Headache	1 of 15	0 of 14
Dizziness	0 of 15	1 of 14

Brain fog	0 of 15	1 of 14
Fatigue	3 of 15	6 of 14
Muscle twitching	1 of 15	0 of 14
Pain, swelling and redness	10 of 15	4 of 14
Pericarditis*	2 of 15	0 of 14
Myocarditis**	0 of 15	1 of 14

*Two controls from the same family developed pericarditis after vaccination
**The PV/PIS who suffered from myocarditis after vaccination repeatedly tested negative for SARS-CoV-2 infection

Persistent symptom(s) duration post-vaccination (≥12 weeks)

Sample group	Number of participants	Percentage (%)
Controls (n=15)	0 of 15	0
PV/PIS (n=14)	14 of 14	100

Participants diagnosed with acute COVID

Note: The percentages shown represent only the subset of participants who experienced an acute COVID-19 infection, with calculations based on this group rather than the total study group.

Sample group	Number of participants	Percentage (%)
**Controls (n=16)**	9 of 16	56.3
Acute COVID pre-vaccination	9 of 9	100
Acute COVID post-vaccination	0 of 9	0
**PV/PIS (n=14)**	8 of 14	57.1
Acute COVID pre-vaccination	4 of 8	50.0
Acute COVID post-vaccination	4 of 8	50.0

WHO severity rating (percentage / %)

Note: The severity rating only represents the subset of participants who experienced an acute COVID-19 infection, with calculations based on this group rather than the total study group.

Level	Number of control participants (n = 9)	Number of PV/PIS participants (n = 8)
Mild	6 of 9	1 of 8
Moderate	1 of 9	4 of 8
Severe	1 of 9	3 of 8
Unknown	1 of 9	0 of 8

(WHO, 2020)

Symptoms experienced during acute COVID infection

Note: This symptom list only represents the subset of participants who experienced an acute COVID-19 infection, with calculations based on this group rather than the total study group.

Symptom	Number of control participants (n = 9)	Number of PV/PIS participants (n = 8)
Asymptomatic	1 of 9	0 of 8
Cough	5 of 9	5 of 8

Sore throat	5 of 9	5 of 8
Fever	5 of 9	7 of 8
Headache	3 of 9	6 of 8
Dizziness	0 of 9	1 of 8
Vomiting	0 of 9	1 of 8
Diarrhoea	0 of 9	1 of 8
Dyspnea (shortness of breath)	1 of 9	1 of 8
Hypertension	1 of 9	2 of 8
Myalgia (muscle pain)	1 of 9	5 of 8
Malaise (general discomfort)	1 of 9	4 of 8
Anosmia (loss of smell)	0 of 9	2 of 8
Dysgeusia (loss of taste)	1 of 9	3 of 8
COVID Type 2 diabetes(2-4)	0 of 9	1 of 8

Participants who developed Long COVID

Note: The percentages shown represent only the subset of participants who experienced an acute COVID-19 infection, with calculations based on this group rather than the total study group.

Cohort that had previous acute COVID infection	Number of participants	Percentage (%)
Controls (n=9)	1 of 9	5.6
PV/PIS (n=8)	4 of 8	50.0

Duration of Long COVID symptoms

Controls (n=9)	>12 weeks
PV/PIS (n=8)	>12 weeks

Symptoms experienced during Long COVID

Symptom	Number of control participants (n = 1)	Number of PV/PIS participants (n = 4)
Fatigue	1 of 1	3 of 4
Brain fog		4 of 4
Heat palpitations		1 of 4
Chest pain		1 of 4
Arthralgia (joint pain)		2 of 4
Digestive problems		1 of 4
Sleep disturbances		2 of 4
Depression/Anxiety		4 of 4

2 Assessing amyloidogenic potential of significant proteins in PV/PIS

Supplementary Table 2. Predicated amyloidogenicity of up and downregulated proteins in PV/PIS relative to controls. This table highlights the potential for proteins to become misfolded and form insoluble amyloid fibrils.

Predicted amyloidogenicity of up and downregulated proteins in PV/PIS relative to controls

Downregulated proteins

Protein Name	Protein ID	Fold change	Amyloid score	Fraction of amyloid residues
Proteins appear in both PV/PIS (n=14) and control (n=16) groups.				
Alpha-1-acid glycoprotein 2	P19652	0.198	0.840	0.338
CD5 antigen-like	O43866	0.283	0.805	0.268
Zinc-alpha-2-glycoprotein	P25311	0.309	0.866	0.235
Keratin, type II cytoskeletal 6B	P04259	0.347	0.863	0.195
Keratin, type II cytoskeletal 3	P12035	0.368	0.863	0.205
Apolipoprotein D	P05090	0.382	0.886	0.249
Transmembrane protein 256	Q8N2U0	0.407	0.747	0.345
Collagen alpha-3 chain	P12111	0.415	0.884	0.256
Protein S100-A9	P06702	0.422	0.550	0.088
Platelet factor 4	P02776	0.433	0.778	0.465
Albumin	P02768	0.435	0.890	0.240
Immunoglobulin lambda-like polypeptide 5	B9A064	0.524	0.722	0.299
Transthyretin	P02766	0.581	0.740	0.306
Keratinocyte differentiation-associated protein	P60985	0.617	0.866	0.283
Immunoglobulin kappa variable 2-28	A0A075B6P5	0.633	0.872	0.467
Keratin, type I cytoskeletal 10	P13645	0.671	0.845	0.149
Keratin, type II cytoskeletal 2 epidermal	P35908	0.680	0.863	0.202
Junction plakoglobin	P14923	0.746	0.881	0.244
Alpha-1-acid glycoprotein 1	P02763	0.752	0.840	0.353
Immunoglobulin kappa light chain	P0DOX7	0.775	0.611	0.173
Keratin, type I cytoskeletal 14	P02533	0.787	0.845	0.100
Keratin, type I cytoskeletal 9	P35527	0.806	0.918	0.122
Keratin, type II cytoskeletal 1	P04264	0.813	0.863	0.188
Keratin, type I cytoskeletal 15	P19012	0.840	0.720	0.151
Vitamin D-binding protein	P02774	0.847	0.899	0.192
Serotransferrin	P02787	0.971	0.779	0.307

Upregulated proteins

Protein Name	Protein ID	Fold change	Amyloid score	Fraction of amyloid residues
Proteins appear in both PV/PIS (n=14) and control (n=16) groups.				

Inter-alpha-trypsin inhibitor heavy chain H4	Q14624	1.07	0.902	0.285
Immunoglobulin heavy variable 5-51	A0A0C4DH38	1.08	0.763	0.513
Plasma protease C1 inhibitor	P05155	1.09	0.866	0.312
Alpha-1-antichymotrypsin	P01011	1.13	0.863	0.338
Serine/threonine-protein phosphatase 2A regulatory subunit B	Q06190	1.13	0.863	0.204
Apolipoprotein M	O95445	1.16	0.822	0.170
Carboxypeptidase B2	Q96IY4	1.18	0.902	0.411
Inter-alpha-trypsin inhibitor heavy chain H3	Q06033	1.28	0.913	0.306
Cholinesterase	P06276	1.31	0.881	0.354
Ficolin-3	O75636	1.33	0.781	0.234
Apolipoprotein C-IV	P55056	1.33	0.696	0.268
Thyroxine-binding globulin	P05543	1.37	0.835	0.287
BPI fold-containing family B member 1	Q8TDL5	1.37	0.918	0.357
Corticosteroid-binding globulin	P08185	1.38	0.853	0.373
Filamin-A	P21333	1.46	0.890	0.190
Prenylcysteine oxidase 1	Q9UHG3	1.52	0.857	0.382
Coagulation factor XI	P03951	1.63	0.881	0.230
Serum amyloid A-2 protein	P0DJI9	1.66	0.845	0.221
Complement factor I	P05156	1.67	0.835	0.290
Serum amyloid A-1 protein	P0DJI8	1.72	0.793	0.230
Plasma serine protease inhibitor	P05154	1.73	0.863	0.288
Immunoglobulin heavy variable 2-26	A0A0B4J1V2	1.78	0.863	0.328
Coagulation factor X	P00742	2.06	0.821	0.232
Tetranectin	P05452	2.31	0.723	0.322
Haptoglobin-related protein	P00739	2.44	0.911	0.262
Attractin	O75882	2.57	0.918	0.276

In this pairwise comparison, fold changes of proteins (or fragments thereof) that were found to be decreased, are denoted as a reciprocal value. Significance was determined at $p<0.05$. Amyloidogenicity was predicted using AmyloGram (http://biongram.biotech.uni.wroc.pl/AmyloGram/, accessed on 19 March 2025).

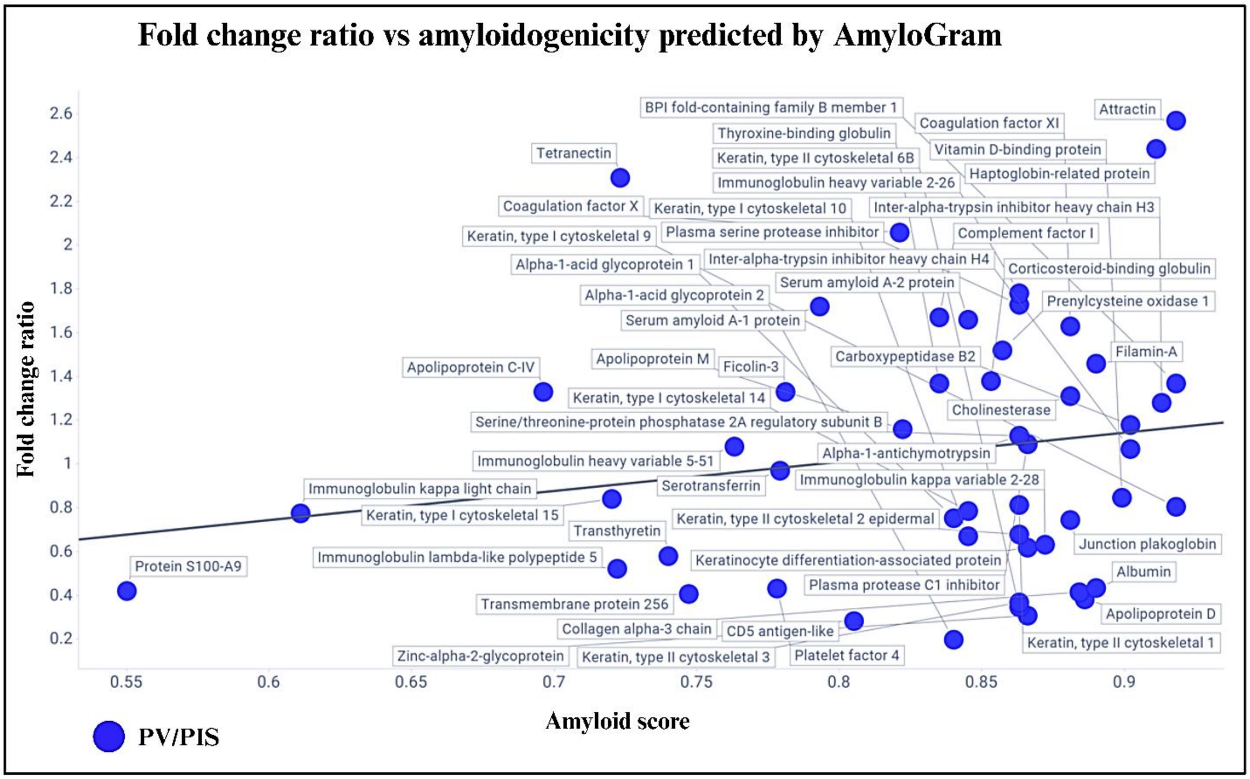

Supplementary Figure 4. Scatter plot illustrating the distribution of signficant proteins and their amyloidogenicty score in PV/PIS pariticipants. The plot highlights no correlation between amyloidogenic potential and signficant protein expression changes in PV/PIS participants – shown by the broad distribution of proteins. Downregulated proteins are denoted by reciprocal values. Amyloidogenicty was predicted using AmyloGram (http://biongram.biotech.uni.wroc.pl/AmyloGram/, accessed on 19 March 2025), and data was plotted using Spotfire® program (http://spotfire.com/, version 12.0, accessed on 14 April 2025).

3 Immune dysregulation, coagulation abnormalities and inflammation

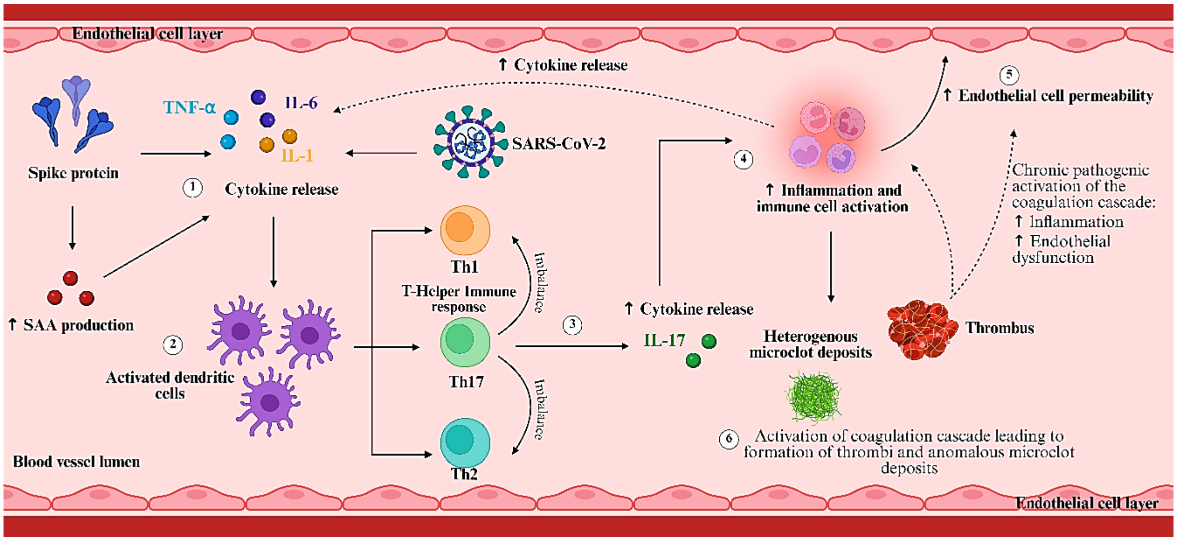

Supplementary Figure 5. The role of T-Helper immune response in PV/PIS. 1) SARS-CoV-2 and spike protein from mRNA vaccines can activate dendritic cells (2), triggering a Th17 immune response and promoting inflammation (3). The upregulation of an inflammatory state (4) can lead to an increase in endothelial permeability (5) resulting in the release of other pro-coagulant factors and adhesion molecules as well as the formation of heterogenous amyloid microclots (6). Created in https://Biorender.com.

4 Comparative overview: Previously described Long COVID dataset (Kruger et al., 2022) vs. PV/PIS

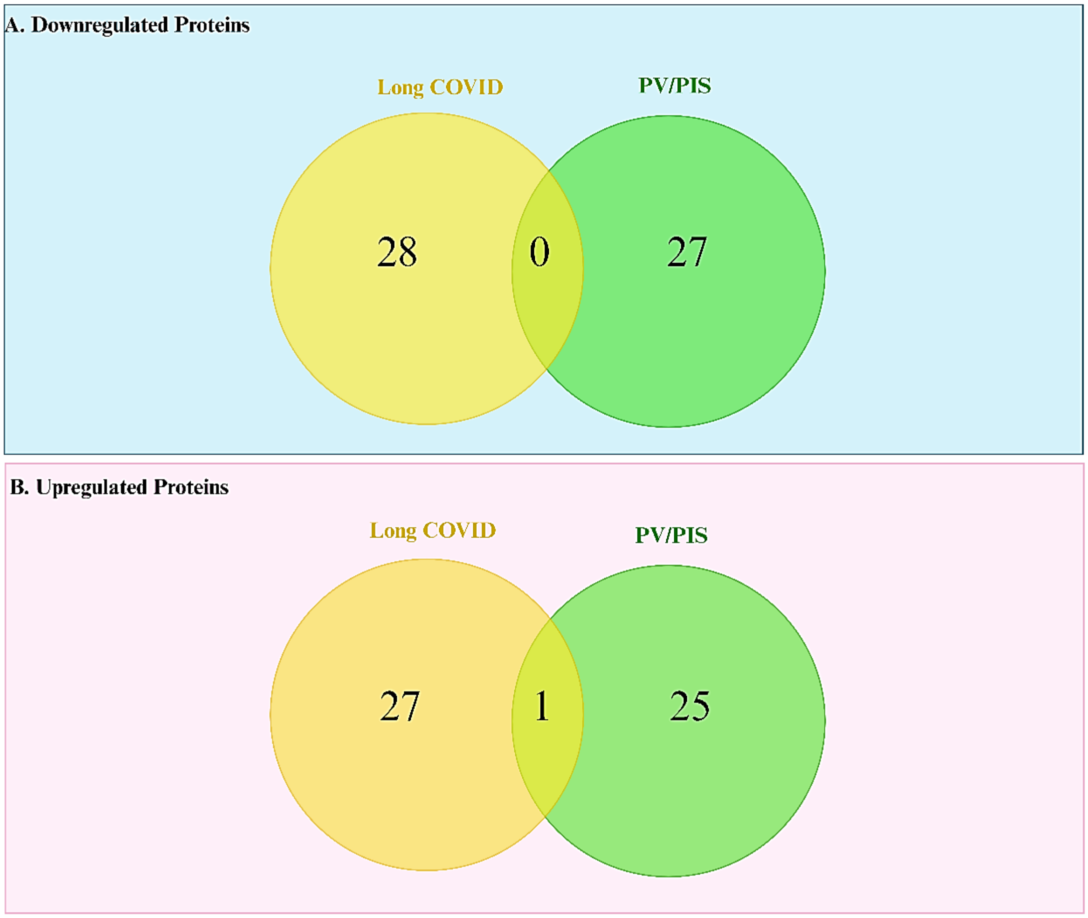

Supplementary Figure 6. Venn diagram illustrating the overlap and differences between the proteins downregulated and upregulated in Long COVID from Kruger et al. (2022) (5), and those unique to PV/PIS in our study. The diagram highlights the number of proteins that were commonly downregulated (A) and upregulated (B) in both conditions, as well as the number of proteins that are uniquely altered in PV/PIS. This comparison provides an overview of the potential distinction in immunological and coagulopathic profiles of Long COVID and PV/PIS. Note, these findings were based on the protein changes listed in the study done by Kruger et al. (2022), vs the protein changes observed in this study. Created in https://Biorender.com.

Supplementary Table 3. Significant protein changes in pairwise analysis of heterogenous amyloid deposits (microclots) from Long COVID relative to controls as previously published by Kruger et al. (2022).

Significant protein changes of Long COVID heterogenous amyloid deposits (microclots) compared to controls

**Downregulated proteins**			
**Protein Name**	**Protein ID**	**Fold Change**	**p-value**
Proteins appear in both Long COVID and control groups			
Mucin-5B	Q9HC84	0.053	<0.001
Immunoglobulin kappa variable 1-12	A0A0C4DH73	0.066	0.026
Prolactin-inducible protein	P12273	0.098	0.026
Immunoglobulin kappa variable 1-6	A0A0C4DH72	0.123	<0.001
Immunoglobulin heavy variable 1-2	P23083	0.130	0.003
Beta-Ala-His dipeptidase	Q96KN2	0.149	<0.001
Histone H4	P62805	0.157	0.023
Immunoglobulin heavy constant alpha 1	P01876	0.177	<0.001
Adiponectin	Q15848	0.202	<0.001
BPI fold-containing family B member 1	Q8TDL5	0.213	<0.001
Hyaluronan-binding protein 2	Q14520	0.222	0.020
Keratin, type II cytoskeletal 6A	P02538	0.261	<0.001
Immunoglobulin lambda variable 3-1	P01715	0.314	<0.001
Keratin, type I cytoskeletal 16	P08779	0.417	<0.001
Immunoglobulin heavy constant alpha 2	P01877	0.421	<0.001
Lactotransferrin	P02788	0.429	0.006
Apolipoprotein C-I	P02654	0.657	0.012
Carboxypeptidase N catalytic chain	P15169	0.658	0.003
**Upregulated proteins**			
**Protein Name**	**Protein ID**	**Fold Change**	**p-value**
Proteins appear in both Long COVID and control groups			
Apolipoprotein C-IV	P55056	1.52	0.018
Immunoglobulin heavy constant alpha 1	P01876	1.53	<0.001
Transforming growth factor-beta-induced protein ig-h3	Q15582	1.70	0.050
Immunoglobulin lambda-like polypeptide 5	B9A064	1.71	<0.001
Immunoglobulin kappa variable 1D-12	P01611	1.73	0.002
Immunoglobulin lambda variable 3-25	P01717	1.78	<0.001
Immunoglobulin lambda-like polypeptide 1	P15814	1.80	0.032
Inter-alpha-trypsin inhibitor heavy chain H4	Q14624	1.89	<0.001
Immunoglobulin heavy variable 3-48	P01763	1.86	0.005
Serum amyloid A-1 protein	P0DJI8	1.96	0.014
Immunoglobulin heavy constant gamma 4	P01861	1.97	<0.001
Immunoglobulin lambda variable 1-40	P01703	2.12	0.014

Immunoglobulin heavy variable 3-23	P01779	2.15	<0.001
Keratin, type I cytoskeletal 16	P08779	2.21	<0.001
Galectin-3-binding protein	Q08380	2.30	<0.001
Thrombospondin-1	P07996	2.36	<0.001
Immunoglobulin lambda variable 7-43	P04211	2.47	0.003
von Willebrand factor	P04275	2.60	<0.001
Immunoglobulin lambda variable 1-51	P01702	2.70	<0.001
Immunoglobulin heavy variable 4-34	P06331	2.75	0.002
Immunoglobulin kappa variable 1-16	P04430	2.75	<0.001
Alpha-1-acid glycoprotein 2	P19652	2.78	<0.001
Selenoprotein P	P49908	2.88	<0.001
Sulfhydryl oxidase 1	O00391	2.90	0.021
Platelet basic protein	P02775	3.53	<0.001
Carbonic anhydrase 1	P00915	3.70	<0.001
Immunoglobulin heavy variable 1-69	P01742	3.80	<0.001
Immunoglobulin lambda variable 3-21	P01719	3.84	<0.001
Immunoglobulin heavy variable 3-33	P01772	4.24	<0.001
Immunoglobulin heavy variable 3-13	P01766	4.31	<0.001
Immunoglobulin kappa variable 1D-16	P01601	4.50	<0.001
Immunoglobulin heavy variable 3-53	P01767	4.96	<0.001
Immunoglobulin heavy variable 2-5	P01817	5.89	0.034
Beta-Ala-His dipeptidase	Q96KN2	6.46	<0.001
Immunoglobulin kappa variable 1-33	P01594	10.33	<0.001
Immunoglobulin kappa variable 2D-28	P06309	20.51	0.013
Immunoglobulin kappa variable 1D-33	P01613	75.99	<0.001

In this pairwise comparison, fold changes of proteins (or fragments thereof) that were found to be decreased, are denoted as a reciprocal value. Significance was determined at $p<0.05$. Long COVID dataset from a previously published study Kruger et al. (2022).

5 References

1. A Minimal Common Outcome Measure Set for Covid-19 Clinical Research. *Lancet Infect Dis* (2020) 20(8):e192-e7. Epub 20200612. doi: 10.1016/s1473-3099(20)30483-7.

2. Gavkare AM, Nanaware N, Rayate AS, Mumbre S, Nagoba BS. Covid-19 Associated Diabetes Mellitus: A Review. *World J Diabetes* (2022) 13(9):729-37. doi: 10.4239/wjd.v13.i9.729.

3. Dallavalasa S, Tulimilli SV, Prakash J, Ramachandra R, Madhunapantula SV, Veeranna RP. Covid-19: Diabetes Perspective-Pathophysiology and Management. *Pathogens* (2023) 12(2). Epub 20230125. doi: 10.3390/pathogens12020184.

4. Miller MG, Terebuh P, Kaelber DC, Xu R, Davis PB. Sars-Cov-2 Infection and New-Onset Type 2 Diabetes among Pediatric Patients, 2020 to 2022. *JAMA Netw Open* (2024) 7(10):e2439444. Epub 20241001. doi: 10.1001/jamanetworkopen.2024.39444.

5. Kruger A, Vlok M, Turner S, Venter C, Laubscher GJ, Kell DB, et al. Proteomics of Fibrin Amyloid Microclots in Long Covid/Post-Acute Sequelae of Covid-19 (Pasc) Shows Many Entrapped Pro-Inflammatory Molecules That May Also Contribute to a Failed Fibrinolytic System. *Cardiovascular Diabetology* (2022) 21(1). doi: 10.1186/s12933-022-01623-4.
