# Supplementary material for: Proteomic signatures of COVID-19 Post-Vaccination/Post-Infection Syndrome (PV/PIS): insights into immune dysregulation and coagulopathy
Source: Front Cell Infect Microbiol. 2026 Mar 25;16:1753348. doi: 10.3389/fcimb.2026.1753348 (PMC13056885; doi:10.3389/fcimb.2026.1753348)
Supplement: Supplementary file 2 [file DataSheet2.pdf]

1.4 Additional participant demographics

Supplementary Table 1. Demographics of participants in both control and PV/PIS groups who experienced acute COVID, including the number of cases that developed Long COVID. This table highlights the complexity of distinguishing between Long COVID and PV/PIS.

Sample demographics

Platelet poor plasma stored previously

Percentage (%) of participants vaccinated

Sample group	Number of participants	Percentage (%)
Controls (n=16)	15 of 16	93.8
PV/PIS (n=14)	14 of 14	100

Type of vaccine administered

Vaccine type and/or combination	Number of control participants (n = 15)	Number of PV/PIS participants (n = 14)
Unknown vaccine information	1 of 15	0 of 14
Pfizer only	9 of 15	14 of 14
J&J only	4 of 15	0 of 14
Pfizer and J&J	1 of 15	0 of 14

Number of vaccinations

Number of vaccine doses received	Number of control participants (n = 15)	Number of PV/PIS participants (n = 14)
Unknown vaccine information	3 of 15	0 of 14
1 dose	4 of 15	4 of 14
2 doses	3 of 15	8 of 14
>2 doses	5 of 15	2 of 14

Symptoms experienced after vaccination

Symptom	Number of control participants (n = 15)	Number of PV/PIS participants (n = 14)
Fever	3 of 15	2 of 14
Chills	2 of 15	0 of 14
Headache	1 of 15	0 of 14
Dizziness	0 of 15	1 of 14
Brain fog	0 of 15	1 of 14
Fatigue	3 of 15	6 of 14
Muscle twitching	1 of 15	0 of 14
Pain, swelling and redness	10 of 15	4 of 14
Pericarditis*	2 of 15	0 of 14
Myocarditis**	0 of 15	1 of 14

*Two controls from the same family developed pericarditis after vaccination

**The PV/PIS who suffered from myocarditis after vaccination repeatedly tested negative for SARS-CoV-2 infection

Persistent symptom(s) duration post-vaccination (≥12 weeks)		
**Sample group**	**Number of participants**	**Percentage (%)**
Controls (n=15)	0 of 15	0
PV/PIS (n=14)	14 of 14	100

Participants diagnosed with acute COVID

Note: The percentages shown represent only the subset of participants who experienced an acute COVID-19 infection, with calculations based on this group rather than the total study group.

Sample group	Number of participants	Percentage (%)
**Controls (n=16)**	9 of 16	56.3
Acute COVID pre-vaccination	9 of 9	100
Acute COVID post-vaccination	0 of 9	0
**PV/PIS (n=14)**	8 of 14	57.1
Acute COVID pre-vaccination	4 of 8	50.0
Acute COVID post-vaccination	4 of 8	50.0

WHO severity rating (percentage / %)

Note: The severity rating only represents the subset of participants who experienced an acute COVID-19 infection, with calculations based on this group rather than the total study group.

Level	Number of control participants (n = 9)	Number of PV/PIS participants (n = 8)
Mild	6 of 9	1 of 8
Moderate	1 of 9	4 of 8
Severe	1 of 9	3 of 8
Unknown	1 of 9	0 of 8

(WHO, 2020) (1)

Symptoms experienced during acute COVID infection

Note: This symptom list only represents the subset of participants who experienced an acute COVID-19 infection, with calculations based on this group rather than the total study group.

Symptom	Number of control participants (n = 9)	Number of PV/PIS participants (n = 8)
Asymptomatic	1 of 9	0 of 8
Cough	5 of 9	5 of 8
Sore throat	5 of 9	5 of 8
Fever	5 of 9	7 of 8
Headache	3 of 9	6 of 8
Dizziness	0 of 9	1 of 8
Vomiting	0 of 9	1 of 8
Diarrhoea	0 of 9	1 of 8
Dyspnea (shortness of breath)	1 of 9	1 of 8
Hypertension	1 of 9	2 of 8
Myalgia (muscle pain)	1 of 9	5 of 8

Malaise (general discomfort)	1 of 9	4 of 8
Anosmia (loss of smell)	0 of 9	2 of 8
Dysgeusia (loss of taste)	1 of 9	3 of 8
COVID Type 2 diabetes(2-4)	0 of 9	1 of 8

Participants who developed Long COVID

Note: The percentages shown represent only the subset of participants who experienced an acute COVID-19 infection, with calculations based on this group rather than the total study group.

Cohort that had previous acute COVID infection	Number of participants	Percentage (%)
Controls (n=9)	1 of 9	5.6
PV/PIS (n=8)	4 of 8	50.0

Duration of Long COVID symptoms

Controls (n=9)	>12 weeks
PV/PIS (n=8)	>12 weeks

Symptoms experienced during Long COVID

Symptom	Number of control participants (n = 1)	Number of PV/PIS participants (n = 4)
Fatigue	1 of 1	3 of 4
Brain fog		4 of 4
Heat palpitations		1 of 4
Chest pain		1 of 4
Arthralgia (joint pain)		2 of 4
Digestive problems		1 of 4
Sleep disturbances		2 of 4
Depression/Anxiety		4 of 4
